# Machine Learning Techniques for Increasing Efficiency of the Robot’s Sensor and Control Information Processing †

**DOI:** 10.3390/s22031062

**Published:** 2022-01-29

**Authors:** Yuriy Kondratenko, Igor Atamanyuk, Ievgen Sidenko, Galyna Kondratenko, Stanislav Sichevskyi

**Affiliations:** 1 Intelligent Information Systems Department, Petro Mohyla Black Sea National University, 68th Desantnykiv Str. 10, 54003 Mykolaiv, Ukraine; ievgen.sidenko@chmnu.edu.ua (I.S.); halyna.kondratenko@chmnu.edu.ua (G.K.); sichevskyi.stanislav@gmail.com (S.S.); 2 Institute of Information Technologies, Warsaw University of Life Science, Nowoursynowska Str. 166, 02-787 Warsaw, Poland; ihor_atamaniuk@sggw.edu.pl; 3 Higher and Applied Mathematics Department, Mykolaiv National Agrarian University, Georgi Gongadze Str. 9, 54020 Mykolaiv, Ukraine

**Keywords:** robotics, sensor, control system, real-time system, machine learning, pattern recognition, classification, fuzzy logic, neural network, canonical decomposition

## Abstract

Real-time systems are widely used in industry, including technological process control systems, industrial automation systems, SCADA systems, testing, and measuring equipment, and robotics. The efficiency of executing an intelligent robot’s mission in many cases depends on the properties of the robot’s sensor and control systems in providing the trajectory planning, recognition of the manipulated objects, adaptation of the desired clamping force of the gripper, obstacle avoidance, and so on. This paper provides an analysis of the approaches and methods for real-time sensor and control information processing with the application of machine learning, as well as successful cases of machine learning application in the synthesis of a robot’s sensor and control systems. Among the robotic systems under investigation are (a) adaptive robots with slip displacement sensors and fuzzy logic implementation for sensor data processing, (b) magnetically controlled mobile robots for moving on inclined and ceiling surfaces with neuro-fuzzy observers and neuro controllers, and (c) robots that are functioning in unknown environments with the prediction of the control system state using statistical learning theory. All obtained results concern the main elements of the two-component robotic system with the mobile robot and adaptive manipulation robot on a fixed base for executing complex missions in non-stationary or uncertain conditions. The design and software implementation stage involves the creation of a structural diagram and description of the selected technologies, training a neural network for recognition and classification of geometric objects, and software implementation of control system components. The Swift programming language is used for the control system design and the CreateML framework is used for creating a neural network. Among the main results are: (a) expanding the capabilities of the intelligent control system by increasing the number of classes for recognition from three (cube, cylinder, and sphere) to five (cube, cylinder, sphere, pyramid, and cone); (b) increasing the validation accuracy (to 100%) for recognition of five different classes using CreateML (YOLOv2 architecture); (c) increasing the training accuracy (to 98.02%) and testing accuracy (to 98.0%) for recognition of five different classes using Torch library (ResNet34 architecture) in less time and number of epochs compared with Create ML (YOLOv2 architecture); (d) increasing the training accuracy (to 99.75%) and testing accuracy (to 99.2%) for recognition of five different classes using Torch library (ResNet34 architecture) and fine-tuning technology; and (e) analyzing the effect of dataset size impact on recognition accuracy with ResNet34 architecture and fine-tuning technology. The results can help to choose efficient (a) design approaches for control robotic devices, (b) machine-learning methods for performing pattern recognition and classification, and (c) computer technologies for designing control systems and simulating robotic devices.

## 1. Introduction

With the development of technology, real-time systems have applications in various fields. Real-time systems are incredibly widely used in industry, including technological process control systems, industrial automation systems, SCADA systems, testing, and measuring equipment, and robotics [1,2,3,4]. Modern technology has increased interest in robotic systems and increased the amount of research conducted in this area. Many studies of such systems in several areas can make human life more manageable. For example, robotics has become an essential technology for the automation industry. Robots ensure maximum accuracy without human error when performing tasks [5].

Modern intellectual robots have high values of dynamic indicators and productively functions under certain modes. The task of robot control is complicated when they work in uncertain environments because robots usually lack full functionality. The supply of robots with efficient remote and tactile sensor systems provides significant functionality and technological capability [3,6].

Intellectual properties are essential for modern robots to gain experience and adapt to natural nonstationary working environments for executing various missions. Service robots acting in uncertain conditions have become even more widely used in recent years [7,8,9,10]. Many modern robots act in clinics, offices, supermarkets, cinemas, enterprises, etc. [11,12,13]. In order for robots to become part of a team and help with tasks in different situations, in particular in dynamic environments inhabited by people, they must move efficiently and without accident [14,15,16] in the target area.

The efficiency of executing an intelligent robot’s mission in many cases depends on the properties of the robot’s sensor and control systems in providing the trajectory planning, recognition of the manipulated objects, adaptation of the desired clamping force of the gripper, obstacle avoidance, and so on (drones, unmanned underwater robots, etc.) [17,18,19,20,21].

To realize the efficient robot’s performance in real-time, particularly in unknown or uncertain environments, the stringent requirements of the robot’s sensor and control systems’ parameters (indicators) must be satisfied. First of all, it concerns:

- Increasing the accuracy of the sensor information of the tactile or remote sensors;
- The minimization of the time of sensor signal formation;
- Decreasing the time of the sensor and control information processing;
- Decreasing the time of the robot’s control system decision-making process in uncertain conditions or a dynamic working environment with obstacles;
- Extending the functional characteristics of the robots based on the implementation of efficient sensors and high-speed calculation algorithms.

Artificial intelligence (AI) methods and algorithms are the perspective tool for designing a robot’s sensors and control systems with improved technical characteristics. Machine learning is a part of artificial intelligence. Machine learning (ML) algorithms build a model based on training data (sample data) for predictions and/or decision-making [22] without implementing traditional programming approaches. ML techniques are used in various applications, such as in image recognition, email filtering, speech recognition, human activity recognition, and computer vision, where it is difficult or unfeasible to develop conventional algorithms to perform the needed tasks [23].

Special attention must be paid to implementing various machine learning algorithms and approaches to robotics because robotics and artificial intelligence, including machine learning, increase and amplify human facilities, enhance productive capacity, and move from simple thinking to human cognitive skills. It opens new opportunities for increasing the efficiency of sensor information processing, recognizing the current situation in the robot working zone, controlling signal processing to realize the desired trajectories, automatic generation of alternative hypotheses, and decision-making in real-time. Among the most popular machine learning algorithm methods and approaches are neural nets, fuzzy sets, fuzzy logic, reinforcement learning, deep learning, semi-supervised learning, time series analysis, unsupervised learning, and regression analysis.

The aim of this work is a development, investigation, and implementation of the different machine learning techniques, including fuzzy logic, neuro systems and networks, and combined neuro-fuzzy approaches and methods of statistical learning theory, for increasing the efficiency of sensor and control information processing in advanced multi-component robotic complexes. Such multi-component robotic complexes (MCRC) are the automatic two-robot systems of the special class that function in non-stationary, uncertain, or unknown working environments. MCRC consists of a moving mobile robot and adaptive robot with a fixed base (manipulator with adaptive gripper) as well as the sensor and control systems. The adaptive robot may be installed on the hull of the moving mobile robot. Mobile robots in MCRC serve as moving motherships for an adaptive robot with a fixed base and can deliver the adaptive robot to any target point of the working surface for executing the corresponding mission. The sensor system of MCRC may consist of various types of tactile sensors, video sensors, and different remote sensors depending on the MCRC missions. High accuracy of manipulation operations, high speed of sensor and control information processing, and high functioning reliability of the mobile and adaptive robots are the main requirements that can be satisfied by the implementation of modern machine learning techniques.

The rest of the article covers multiple aspects related to the topic discussion. Section 2 deals with the analysis of published related works and formulation of the problem statement. Section 3 covers a general representation of the proposed fuzzy information processing technique for the adaptive robot’s sensor system detecting the slip displacement signal and recognizing the direction of the unknown object slippage. Section 4 presents a neuro-fuzzy observer of clamping force and a neuro-controller for the control system of the mobile robot, which can move on inclined and vertical ferromagnetic surfaces. In Section 5, the authors provide a detailed description of the prediction procedure for providing reliable functioning MCRS, including robot sensor and control systems, based on the canonical decomposition of the statistical data. Section 6 and Section 7 deal with the implementation of the open-source software for designing the adaptive robot’s control system with the training of a convolution neural network for the recognition of the object shape in the working zone of the robot based on video-sensor information. The paper ends with a conclusion in Section 8.

## 2. Related Works and Problem Statement

In recent years, the role of machine learning has significantly increased. Machine learning techniques have many successful applications in different areas of human activity, in particular, in medicine [4,24,25,26,27,28,29,30,31], agriculture [32,33,34,35,36], transportation [37,38,39,40,41,42], energy production [43,44], finance markets [45], investment policy [46] and research [47]. Statistical learning theory is efficiently used for processing data from sensors in real-time based on effective multi-output Gaussian processes [48] and for prognosis of the state of technical objects using canonical decomposition of a random sequence [49].

Let us analyze the peculiarities of ML techniques’ implementation in robotics as this article is devoted to the increasing efficiency of robot’s sensor and control information processing using appropriate machine learning algorithms.

Machine learning techniques are successfully implemented for robot control with model-based reinforcement learning [50], for convergence machine learning methods and robotics in co-assembly [51], for intelligent and autonomous surgical robotics [28], for speed control when creating robots of the type “leader-follower” with the application of fuzzy sets and fuzzy logic, and supervised machine learning [52], for computer-aided design based on machine learning for space research and control of autonomous aerial robots [53], and for robotics and automation using simulation-driven machine learning [54].

### 2.1. Machine Learning Techniques for Robotics in Industrial Automation

Rajawat et al., in [55], introduce a newer approach to process automation using robotic devices, which increases efficiency and product quality with the application of artificial intelligence and machine learning techniques by the implementation of control and repetitiveness of robotics with human flexibility and functionality. The paper [56] discusses an application of the ML methods (random forest, artificial neural network) to accurately model surface roughness in wire arc additive manufacturing. In [57], Wang et al., especially for the robotic assembly system, propose an image processing method based on machine learning algorithms. Mayr et al. discuss the optimizing linear winding process in electric motor manufacturing based on machine learning techniques and sensor integration [58]. Al-Mousawi, in [59], synthesizes the detection system for magnetic explosives based on machine learning techniques and a wireless sensor network. Martins et al., in [60], consider the application of the machine learning techniques for cognitive robotic process automation. Segreto et al., propose to use different machine learning techniques for in-process end-point detection in robot-assisted polishing using multiple sensor monitoring [61].

### 2.2. Machine Learning in Robot Path Planning and Control

The approach (with application to mobile robots), presented in [62], uses machine learning techniques to improve the connection between low-level and high-level representations of sensing and planning, respectively. Qijie et al., propose [21] a planning path algorithm for mobile robots in unknown and uncertain environments based on rapidly exploring random trees and reinforcement learning SARSA (λ). The article [63] concerns exoskeleton robot applications and presents various data modes as input parameters to models of machine learning to increase the timeliness, motion accuracy, and safety of gait planning. In [64], the authors provide a review of deep learning and robotic capture, as well as tracking and gait planning problems. The basketball-training robot provides intelligent path autonomous planning and approaches the target point by avoiding obstacles [65]. The robot path planning approach is described in [20] with the implementation of the deep reinforcement learning method.

### 2.3. Machine Learning for Information Processing in Robot Tactile and Remote Sensors

The review in [66] researches the combination of electronic skins and machine learning techniques. The authors demonstrate how researchers can use the latest developments from the above two areas to create autonomous robots with deployable functions. They were integrated with informative sensory and proprioceptive capabilities to face complex conditions in real situations. Ibrahim et al. present embedded machine learning methods [67] for near sensors tactile data processing. Keser et al. use ML techniques for surface roughness recognition based on fiber optic tactile sensor data [68]. In [69], ML regression algorithms are trained based on proprioceptive sensing for predicting slippage of individual wheels in off-road mobile robots. Wei et al. propose a fusion method with the application of support vector machine and evidence theory for robot target detection and recognition using multi-sensor information processing [70]. Martinez-Hernandez et al. use a tactile robot for autonomous and adaptive exploration of object shape using learning from sensory predictions [71]. A smart capacitive sensor skin with embedded data quality indication for enhanced safety in human–robot interaction is proposed in [72] with the implementation of two ML algorithms, in particular, a neural network and a support vector machine.

### 2.4. Machine Learning in Robot Computer Vision

Joshi et al., in [73], demonstrate a method based on deep reinforcement learning to solve a robotic gripping problem using visio-motor feedback. A posture assessment system based on the “eye-to-hand” camera has been developed in [74] for robotic machining, and the accuracy of the estimated pose is improved using two different approaches, namely sparse regression, and LSTM neural networks. Inoue et al. propose [19] a machine vision approach for robots with autonomous navigation based on a stereo camera and convolutional neural networks (deep learning technique) for the avoidance of obstacles. Mishra et al. consider robotic vision solutions for pedestrian detection in the working zone of mobile robots based on deep learning techniques [75].

### 2.5. Machine Learning for Increasing Reliability and Fault Diagnostics

To increase the productivity of automated industrial processes, it is necessary to monitor and estimate the current state of the robots and manufacturing equipment. Long et al. use attitude data for intelligent fault diagnosis of multi-joint industrial robots [76] based on a deep hybrid learning structure (sparse auto-encoder and support vector machine). Subha et al. consider the problem of sensor fault diagnostics for autonomous underwater robots using an extreme learning machine [77]. Severo de Souza, in [78], considers increasing the reliability of the production system by detecting abnormal sensors based on machine learning techniques and information from the wireless sensor network. In [2], a predictive maintenance system for production lines in manufacturing can detect signals for potential failures before they occur based on the real-time application of the IoT data and machine learning techniques. Kamizono et al. in [79] propose a fault detection and classification approach based on a neural network with a harmonic sensor for preventing a robotic error.

The above-discussed analysis of last publications on the implementation of machine learning in robotics shows that researchers continue to improve machine learning techniques [47,80,81] and develop new machine learning solutions [40,82,83,84,85] for intelligent robots using well-known and new design methods, approaches, and methodologies. It deals, first, with the new and specific missions of the ground, underwater, and aerial mobile robots, as well as with the high level of information uncertainty concerning the nature of the robot environment, changing character of manipulated object parameters, and unknown behavior of the dynamic obstacles. Additional design requirements also stimulate research into the development of new approaches for the implementation of machine learning methods and algorithms in modern robotics.

Thus, developing new design methods, algorithms, and models is reasonably necessary to provide efficient sensor and control information processing. It will simultaneously improve the design processes for robot navigation-control systems and increase the control indexes of their functioning in uncertain environments.

The problem statement of this article deals with the implementation and investigation of advanced ML techniques based on the fuzzy sets theory, theory of neuro system, statistical learning theory, and others, for the increasing efficiency of robot sensor and control information processing in multi-component robotic complexes that function in non-stationary, uncertain, or unknown working environments. The missions of the considered MCRC may deal with the preliminary unknown or changeable mass of the manipulated objects and with the need to avoid obstacles or to correct the adaptive robot’s path in collisions with obstacles [3,6,7,86,87,88,89,90,91,92,93]. In this case, the adaptive robot with a fixed base should have the possibility to identify object mass and directions of the manipulated object slippage. Such adaptive robots may be equipped with different tactile sensors and can work separately or may be installed at the moving mothership mobile robot as the second robotic component of MCMC. For mobile robots which can move on inclined, vertical, or ceiling ferromagnetic surfaces (ship hulls, for example), it is very important to ensure high indicators of control and maneuvering characteristics and to provide the required magnetic clamping force between the mobile robot and working surface [13,15,17,94,95,96,97,98]. The mobile robot should track the working surface parameters and create a reliable value of the clamping force for high-reliable MCRC functioning in the action of surface disturbances, taking into account that a ferromagnetic surface may be covered by nonmagnetic layers of dead microorganisms. In these cases, the size of the gap may have a non-stationary character. It is necessary also, to keep and support MCRC’s high functionality [34,49,84] by controlling and predicting the technical state of all MCRC components. With the regular operation of MCRC, it is necessary to evaluate in real-time the operability of the corresponding devices for sensor and control information processing, to ensure their continuous operation and predict possible failures. For many important MCRC missions, real-time object recognition can be provided by using the video camera on the manipulator’s arm. In these cases, the recognized objects should be classified, and the images should be transmitted to the control panel, for example, a mobile phone. Creating the ML models of neural networks with YOLOv2 and ResNet34 architectures is a prospective approach for the recognition and classification of the different objects in images. For the development of the optimal structure of the MCRC’s control system during design processes, it is necessary to implement a simulation approach based on a MoveIt environment that allows the obtaining of configuration files of the manipulator’s arm and transferring them to the MCRC’s control system.

Finally, let us formulate the aims of this research as:

- Implementing the machine learning algorithms for extension of functional features of adaptive robots; in particular, using fuzzy and neuro net approaches for sensor information processing within the recognition of the slippage direction of manipulated objects in the robot gripper during its contact with the obstacles;
- Approximating the “clamping force—air gap” nonstationary functional dependence based on a neuro-fuzzy technique for the mobile robot control system, which provides increased reliability for robot movement on inclined electromagnetic surfaces;
- Implementing the statistical learning theory for increasing the efficiency of a robot’s sensor system based on the developed algorithms of prediction control;
- Developing the machine learning models and corresponding software for recognizing manipulated objects [99] using video–sensor information processing with a discussion of the peculiarities of the convolutional-neural network’s training process.

## 3. The Machine Learning Algorithms for Extension of Functional Properties of Adaptive Robots with Slip Displacement Sensors

One of the efficient approaches to determine the unknown mass of a manipulated object and the desired value of clamping force is the use of tactile sensors that provide detection of the object slippage between the gripper fingers [3,6,12,86,87,88,89,90,91,92,93,100,101,102]. Besides this, slip displacement information can be used to recognize objects from a set of alternatives and for correction of the control algorithm and robot gripper’s trajectory. The design process for the slip displacement sensors (SDS) is based on the implementation of different detection methods [89,92,103], including rolling motion, vibration, or changing a configuration of sensitive elements, the friction registration, oscillation of the circuit parameters, displacement of the fixed sensitive elements, and others.

Let us discuss the task of tactile sensor information processing based on machine learning algorithms to recognize the object slippage and slippage direction (in the gripper of the adaptive robot).

Most SDS form the sensor information for the robot control system only about slippage as an event (1—true, 0—false). An additional problem is identifying slippage direction, which is very important for situations when a robot gripper contacts an unknown obstacle in the robot working zone [89,103,104]. In many cases, the appearance of the obstacles has random character. In collision situations, the slippage direction depends on the robot’s gripper trajectory and coordinates of the obstacles in the adaptive robot’s working space.

It is possible to acquire information about slippage direction using (a) multi-component slip displacement sensors and (b) the machine learning algorithms (fuzzy logic, neuro networks) for sensor information processing. Multi-component slip displacement sensors can detect the sensitive rod displacement in the special cavity using a group of Hall sensors [90], capacitive sensors [89,102,103], or resistance sensors based on the electro-conductive rubber [88].

**Figure 1:**
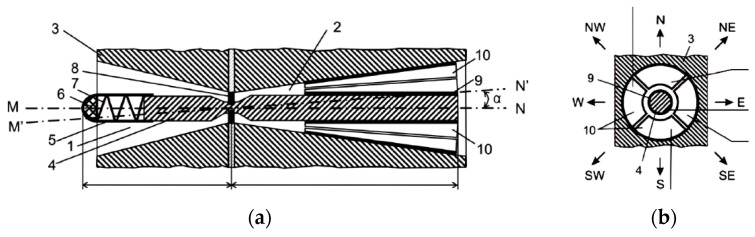
Capacitive slip displacement sensor with two-part sensitive rod: (**a**) front view; (**b**) right view: 1, 2—first and second cavities of the robot’s finger; 3—the robot’s finger; 4—two-part rod (sensitive element); 5—elastic tip; 6—elastic contact surface; 7—spring; 8—resilient element; 9, 10—multi-component capacitor plates.

Let us consider a fuzzy logic approach for identifying slippage direction based on the capacitive slip displacement sensor [89,103], presented in Figure 1.

The SDS is placed on at least one of the gripper fingers (Figure 1). The recording element consists of four capacitors distributed across the conical surface of the special cavity (2). One plate (9) of each capacitor is located on the rod (4) surface, and the second plate (10) on the inner surface of the cavity (2).

**Figure 2:**
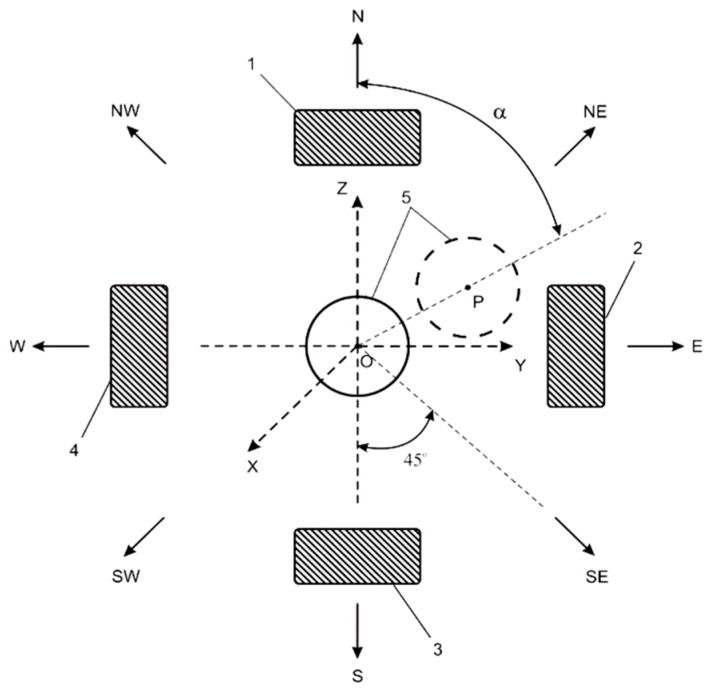
Sensor for recognition of slippage direction: 1, 2, 3, 4—capacitors *C*_1_, *C*_2_, *C*_3_, and *C*_4_; 5—deviating rod; point O—initial position of the sensitive rod before object slippage; point P—final position of the sensitive rod after object slippage; N (0°/360°), NE (45°), E (90°), SE (135°), S (180°), SW (225°), W (270°), NW (315°)—directions of the object slippage; OXYZ—coordinates system for the robot’s working space; OX—direction for creating clamping force between gripper fingers; α—slippage direction.

The sensitive element 4 will move to the direction {N, NE, E, SE, S, SW, W, NW} and intermediate positions, for example, from point O to point P, depending on the corresponding direction of the object slippage in the robot gripper in cases of contacting obstacles (Figure 2).

Reciprocal movements of plates (9) and (10) in all capacitive elements lead to value changes of the capacities *C*_1_, *C*_2_, *C*_3_, and *C*_4_, depending on the direction of the rod’s movement.

Using some experimental data for different displacement directions and corresponding changes of the capacities *C*_1_, *C*_2_, *C*_3_, and *C*_4_, it is possible to build and learn a corresponding neural network with four input signals (*C*_1_, *C*_2_, *C*_3_, *C*_4_) and one output signal (direction *α* within the interval [0°, 360°], fuzzy rules.

Another machine learning approach based on the fuzzy logic implementation [38,103,105] can be realized based on the set of adjustments of the rule consequents using the above mentioned experimental data or simulation results.

**Figure 3:**
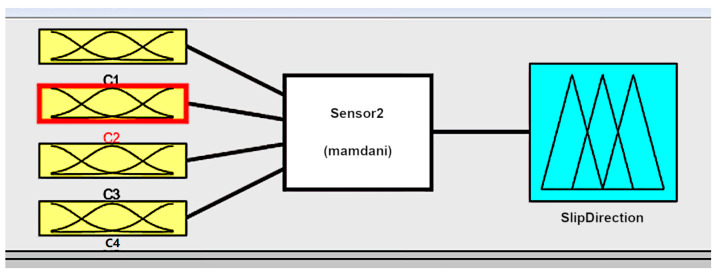
The structure of the fuzzy system with 3 linguistic terms for input signals and 9 linguistic terms for the output signal.

**Figure 4:**
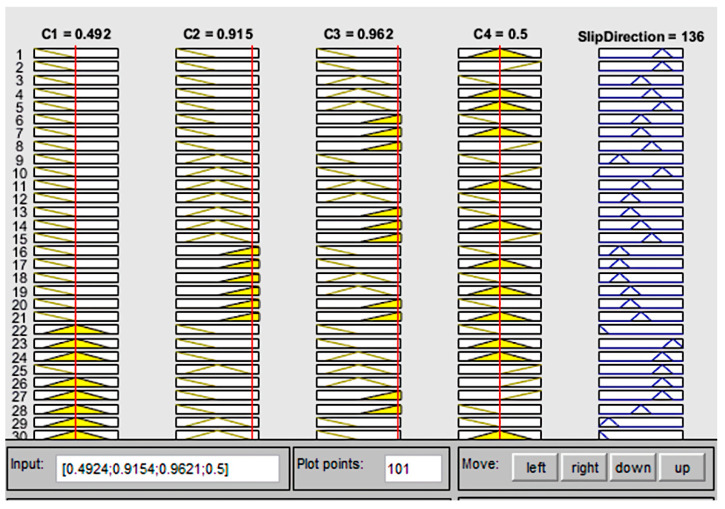
The fragment of the fuzzy rule base for the identification of the slippage direction.

The structure of the fuzzy system (FS) of the Mamdani type [106] for slip displacement sensor information processing is presented in Figure 3 and a fragment of fuzzy rule base is presented in Figure 4.

Each fuzzy rule in the fuzzy rule base has the following structure:

IF (Condition–Antecedent), THEN (Result–Consequent),(1)

which deals with the determination of the dependence:

(2) $$\alpha =f_{FS}\left(C_{1},C_{2},C_{3},C_{4}\right),$$

between slip displacement direction *α* and capacitive values *C*_1_, *C*_2_, *C*_3_, *C*_4_.

*C*_1_, *C*_2_, *C*_3_, and *C*_4_ are output signals of corresponding capacitor sensitive components, and simultaneously input signals for the designed fuzzy system.

**Figure 5:**
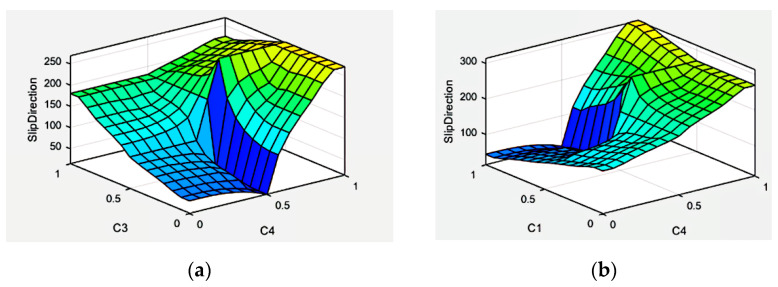
Characteristic surfaces of the fuzzy system $\alpha =f_{FS}\left(C_{1},C_{2},C_{3},C_{4}\right)$: (**a**) *C*_1_ = const; *C*_2_ = const; (**b**) *C*_2_ = const; *C*_3_ = const.

The characterized surfaces of the designed fuzzy system of the Mamdani type for slip displacement sensor information processing are presented in Figure 5.

Simulation results show that the designed fuzzy system provides efficient sensor information processing and can calculate the slippage direction for any natural combinations of the measured capacitive parameters *C*_1_, *C*_2_, *C*_3_, *C*_4_, which correspond to the displacement of the sensitive element (rod 4 in Figure 1).

The application of the fuzzy logic method of sensor information processing allows the expanding of the functional properties of the adaptive robot with the possibility to correct the trajectory for obstacle avoidance.

It is possible to improve the quality of the designed fuzzy system and increase the efficiency of sensor information processing by using different structural and parametric optimization methods for fuzzy systems [107,108,109,110,111,112].

The novelty of the presented results consists of (a) the developed structure and intelligent rule base of the fuzzy system for sensor information processing during the slip displacement detection and recognition of the object’s slippage direction within the interval of 0–360 degrees as well as (b) developed ML algorithm and information communications of the proposed fuzzy system and the original capacitive multi-component slip displacement sensor. Patent of Ukraine (Patent No. 52080) defends the engineering solution of the considered robot’s slip displacement sensor (Figure 1).

## 4. Neuro-Fuzzy Techniques in Control Systems of Mobile Robots That Can Move the Operation Tool on Inclined, Vertical, and Ceiling Ferromagnetic Surfaces

In the modern world, there are particular needs for mobile robots (MR) to move on different horizontal, inclined, or vertical surfaces. MR can use various types of propelling and pressure devices, for example, for cleaning the exterior parts of ships and other constructures afloat or in a dry dock when automating processes in shipbuilding [98,113,114,115]. Such robots can perform various complex, resource-intensive, and hazardous to people’s lives and health, works. For example, cleaning large vertical surfaces and hard-to-reach places, decontamination under radiation conditions, installation of dowels and explosive devices, firefighting, painting, inspection, diagnostics, etc.

**Figure 6:**
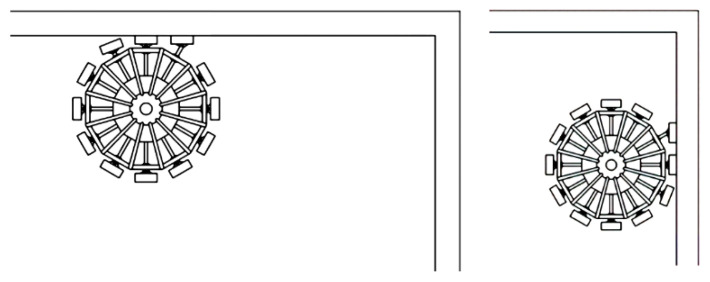
Two intermediate states of the magnet-controlled wheel of mobile robots with different positions of the stepping legs: the movement on the ceiling (**left**) and vertical (**right**) electro-conductive surfaces.

**Figure 7:**
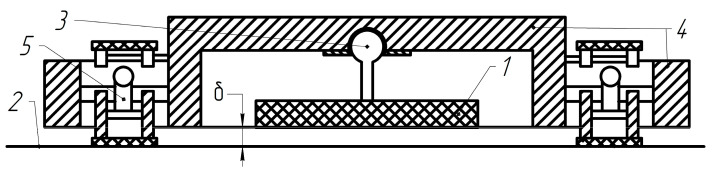
Multipurpose caterpillar MR: 1—main clamping magnet; 2—ferromagnetic surface; 3—spherical joint 3; 4—frame; 5—right and left tracks; δ—clearance.

An important element in the control of such robots is to ensure reliable adhesion (gripping) of the MR to the surface and its retention without slipping when performing various tasks [98]. Clamping devices with magnetic fastening provide effective grip to the surface using electromagnets. Mobile robots, presented in Figure 6 and Figure 7, improve the performance and reliability of technological operations on ferromagnetic surfaces [15,17,98,116,117].

**Figure 8:**
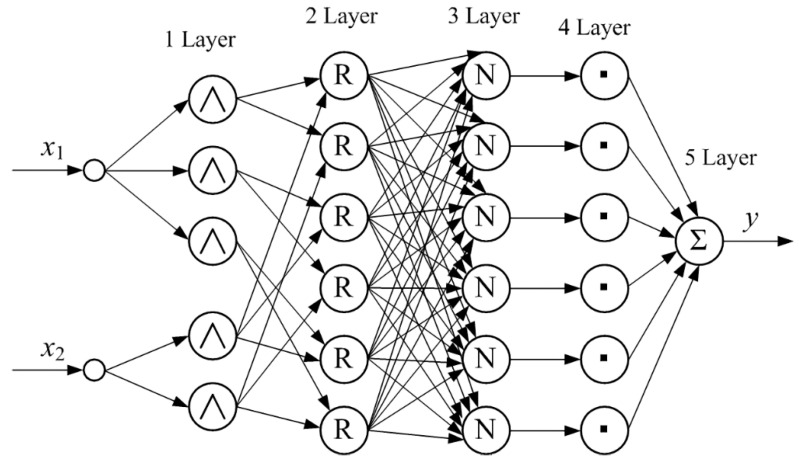
Functional structure of typical ANFIS with two inputs *x*_1_, *x*_2_, and one output *y*.

Machine learning methods based on the adaptive neuro-fuzzy inference engine can be successfully used for the synthesis of the clamping force observer [17,98] for MR, presented in Figure 6. The adaptive network-based fuzzy inference system (ANFIS) is an artificial neural network based on a fuzzy inference system (FIS) [98]. ANFIS presents in the form of the neural network with five layers and the forward signal propagation (Figure 8). Each node at the first ANFIS layer corresponds to the linguistic term (LT) of a certain input signal. Thus, the total number of nodes is equal to the sum of all LTs for all input signals. ANFIS in Figure 8 has two LTs (Small, Large) and three LTs (Small, Middle, Large) for first and second input signals, and a total of five nodes at the first ANFIS layer. The second, third, and fourth layers of the ANFIS consist of the six nodes according to the number of fuzzy rules, degrees of antecedent realization, and contributions of the corresponding fuzzy rules. Such contributions are summarized in one node of the fifth ANFIS layer to form a resulting output signal.

ANFIS allows improving sensor information processing based on the measurement of the gap between the robot’s clamping magnet and non-stationary ferromagnetic surface covered by non-ferromagnetic components. The shape of linguistic terms’ membership functions of the input variables significantly affects the training process of the ANFIS and clamping force observer accuracy.

**Figure 9:**
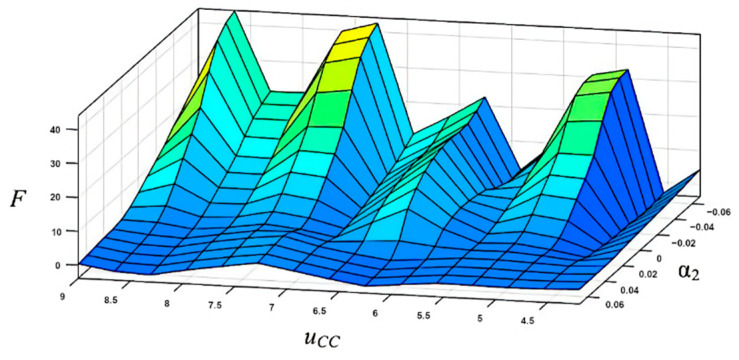
Characteristic surface based on Gaussian-2 membership functions.

The comparative results demonstrate the high accuracy of the desired clamping force calculation by the developed fuzzy-neuro observer. In particular, the training error 0.187 of ANFIS with Gaussian 2 membership functions for the linguistic terms of its input signals is less in 1.23 and 2.75 times, compared with using π-like and trapezoidal membership functions, correspondingly. The ANFIS characteristic surface for the input variable linguistic terms with Gaussian 2 membership functions is presented in Figure 9.

Successful cases for improving control information processing deal with the implementation of fuzzy and neuro controllers for mobile robot control in uncertain or unknown environments. For example, investigation of the machine learning algorithms [116,117] for mobile caterpillar robots (Figure 7) demonstrated the high efficiency of the neural controllers’ implementation for control of mobile robot movement on the desired trajectory. The synthesis procedure of the designed controllers is developed using a genetic algorithm. The fitness function is based on the control quality of two output signals, in particular, speed and angle. The comparative results demonstrate the high efficiency of the proposed machine learning technique, in particular, transient times for the MR control system with neuro controllers compared with conventional PID-controllers (with optimal parameters) are decreased by 2.2 times for the MR’s speed control channel and by 1.23 times for the MR’s angle control channel.

The novelty of the presented results deals with the application of machine learning techniques for original constructions of the magnetically wheel-controlled mobile robot (Figure 6) with the neuro-fuzzy observer of clamping force, and caterpillar mobile robot (Figure 7) with neuro controller for improving sensor and control information processing. Patents of Ukraine (Patent No. 45369, Patent No. 47369, Patent No. 100341) defend considered engineering solutions for mobile robots.

## 5. Prediction Control of Robot Sensor and Control Systems Based on the Canonical Decomposition of the Statistical Data

The reason for sensor system errors is due to the natural wear of the sensors, as well as the peculiarities of their functioning [118,119,120,121,122]:

- Critical operating conditions (high/low temperature, humidity, pressure, pollution, illumination, etc.);
- The autonomy of work;
- Changing the mutual orientation of the sensor and the recognition object;
- Work in real-time (almost always);
- Limited resources.

Errors in sensor systems significantly reduce the quality of operation of robot control systems, which can lead to catastrophic consequences (at critical infrastructure facilities). In this regard, the estimation of the operability of the control system in real-time [4,123] is an important and urgent task, taking into account the peculiarities of changing the operating conditions and their investigations based on machine learning.

To ensure high reliability of the operation of control systems, it is proposed to use the predictive control [124,125] module in the general structure—an estimation of the system’s state at future points in time, followed by a decision on the suitability of its use. In the general case, the parameter S, characterizing the quality of the system’s functioning (time of operation, the number of operations per unit of time, the accuracy of the operation, etc.) is random. Therefore, to solve the problem of estimation of the system’s future state, it is necessary to use the methods of the theory of random functions and random sequences. The canonical decomposition of a random sequence $\left\{S\right\}=S\left(i\right),i=\bar{1,I}$ of the changeable parameter S at the moments in time $t_{i},\;i=\bar{1,I}$ is the most universal (from the point of view of restrictions) mathematical model [120]:

(3) $$S\left(i\right)=E\left[S\left(i\right)\right]+\sum_{\nu =1}^{i}\sum_{\lambda =1}^{N}P_{\nu }^{\left(\lambda \right)}\rho \left(\lambda ,\nu ;1,i\right),\;i=\bar{1,I},$$

where E[] is a mathematical expectation.

The elements of the canonical expansion $P_{\nu }^{\left(\lambda \right)},\;\rho \left(\lambda ,\nu ;h,i\right)$ are determined by the recurrent relations [120,124]:

(4) $$\begin{array}{c} P_{\nu }^{\left(\lambda \right)}=S^{\lambda }\left(\nu \right)-E\left[S^{\lambda }\left(\nu \right)\right]-\sum_{\mu =1}^{\nu -1}\sum_{j=1}^{N}P_{\mu }^{\left(j\right)}\rho \left(j,\mu ;\lambda ,\nu \right)-\\ -\sum_{j=1}^{\lambda -1}P_{\nu }^{\left(j\right)}\rho \left(j,\nu ;\lambda ,\nu \right),\;\lambda =\bar{1,N},\nu =\bar{1,I}; \end{array}$$

(5) $$\rho \left(\lambda ,\nu ;h,i\right)=E\left[P_{\nu }^{\left(\lambda \right)}\left(S^{h}\left(i\right)-E\left[S^{h}\left(i\right)\right]\right)\right]/E\left[{\left\{P_{\nu }^{\left(\lambda \right)}\right\}}^{2}\right]=\;\frac{1}{D_{\lambda }\left(\nu \right)}\{E\left[S^{\lambda }\left(\nu \right)S^{h}\left(i\right)\right]-E\left[S^{\lambda }\left(\nu \right)\right]E\left[S^{h}\left(i\right)\right]-\;-\sum_{\mu =1}^{\nu -1}\sum_{j=1}^{N}D_{j}\left(\mu \right)\rho \left(j,\mu ;\lambda ,\nu \right)\rho \left(j,\mu ;h,i\right)-\;-\sum_{j=1}^{\lambda -1}D_{j}\left(\nu \right)\rho \left(j,\nu ;\lambda ,\nu \right)\rho \left(j,\nu ;h,i\right)\},\;\lambda =\bar{1,h},\;\nu =\bar{1,i},\;h=\bar{1,N},\;i=\bar{1,I};$$

(6) $$\begin{array}{c} D_{\lambda }\left(\nu \right)=E\left[{\left\{P_{\nu }^{\left(\lambda \right)}\right\}}^{2}\right]=M\left[S^{2\lambda }\left(\nu \right)\right]-M^{2}\left[S^{\lambda }\left(\nu \right)\right]-\\ -\sum_{\mu =1}^{\nu -1}\sum_{j=1}^{N}D_{j}\left(\mu \right){\left\{\rho \left(j,\mu ;\lambda ,\nu \right)\right\}}^{2}-\sum_{j=1}^{\lambda -1}D_{j}\left(\nu \right){\left\{\rho \left(j,\nu ;\lambda ,\nu \right)\right\}}^{2},\;\lambda =\bar{1,N},\nu =\bar{1,I}. \end{array}$$

The coordinate functions $\rho \left(\lambda ,\nu ;h,i\right),\;\nu =\bar{1,i};\;\lambda ,h=\bar{1,N};\;i=\bar{1,I}$ are characterized by the properties:

$$\rho \left(\lambda ,\nu ;h,i\right)=\left\{ \begin{array}{l} 1,\;\mathrm{if}\;\left(h=\lambda \right)\wedge \left(\nu =i\right);\\ 0,\;\mathrm{for}\;\left(i<\nu \right)\vee \left(\left(h<\lambda \right)\wedge \left(\nu =i\right)\right). \end{array}\right.\;$$

Nonlinear model (1) of the random sequence $\left\{S\right\}=S\left(i\right),\;i=\bar{1,I}$ contains N arrays $\left\{P^{\left(\lambda \right)}\right\},\;\lambda =\bar{1,N}$ of uncorrelated centered random coefficients $P_{i}^{\left(\lambda \right)},\;\lambda =\bar{1,N},i=\bar{1,I}.$ Each of these coefficients contains information about the corresponding value $S^{\lambda }(i),\;\lambda =\bar{1,N},\;i=\bar{1,I}$, and the coordinate functions $\rho \left(\lambda ,\nu ;h,i\right),\;\nu =\bar{1,i},\;\lambda ,h=\bar{1,N},\;i=\bar{1,I}$ describe probabilistic relations of the order λ+h between the sections $t_{\nu }$ and $t_{i},\;\left(\nu ,i=\bar{1,I}\right).$

Sequential substitution of values $s\left(\nu \right),\;\nu =\bar{1,k}$ into an expression (1) with the subsequent application of the mathematical expectation operation allows one to obtain an estimate $\hat{s}\left(i\right),\;i=\bar{k+1,I}$ of the investigated parameter at future points in time $t_{i},\;i=\bar{k+1,I}$:

(7) $${\hat{s}}_{}^{\left(\mu ,l\right)}\left(h,i\right)=\{\begin{array}{c} E\left[S^{h}\left(i\right)\right],\;for\;\mu =0\\ {\hat{s}}_{}^{\left(\mu ,l-1\right)}\left(h,i\right)+\left(s^{l}\left(\mu \right)-{\hat{s}}_{}^{\left(\mu ,l-1\right)}\left(l,\mu \right)\right)\rho \left(l,\mu ;h,i\right),\;for\;l\neq 1\\ {\hat{s}}_{}^{\left(\mu -1,N\right)}\left(h,i\right)+\left(s^{l}\left(\mu \right)-{\hat{s}}_{}^{\left(\mu -1,N\right)}\left(l,\mu \right)\right)\rho \left(l,\mu ;h,i\right),\;for\;l=1 \end{array},$$

where $\;\hat{s}{\;}_{}^{\left(\mu ,l\right)}\left(h,i\right)=E\left[s^{h}\left(i\right)/s^{\nu }\left(j\right)\right]:\;(a)\;\nu =\bar{1,N},\;for\;j=\bar{1,\mu -1};\;(b)\;\nu =\bar{1,l},\;for\;j=\mu$ is an optimal estimate of the future value $s^{h}\left(i\right)$ according to the minimum mean square of the extrapolation error criterion.

The predictive model (5) can be converted to:

(8) $${\hat{s}}_{}^{\left(k,N\right)}\left(1,i\right)=E\left[S\left(i\right)\right]+\sum_{j=1}^{k}\sum_{\nu =1}^{N}\left(s^{\nu }\left(j\right)-E\left[S^{\nu }\left(j\right)\right]\right)T_{\left((j-1)N+\nu \right)}^{\left(kN\right)}\left(\left(i-1\right)N+1\right),$$

where:

(9) $$T_{\lambda }^{\left(\alpha \right)}\left(\xi \right)=\left\{ \begin{array}{l} T_{\lambda }^{\left(\alpha -1\right)}\left(\xi \right)-T_{\lambda }^{\left(\alpha -1\right)}\left(\alpha \right)\tau_{k}\left(i\right),\;\mathrm{if}\;\lambda \leq \alpha -1;\\ \gamma_{\alpha }\left(\xi \right),\;\mathrm{for}\;\lambda =\alpha ; \end{array}\right.$$

(10) $$\tau_{\alpha }\left(\xi \right)=\left\{ \begin{array}{l} \rho \left({\mathrm{mod}}_{N}(\alpha ),[\alpha /N]+1;1,\left[\alpha /N\right]+1\right),\;\mathrm{for}\;\xi \leq kN;\\ \rho \left({\mathrm{mod}}_{N}(\alpha ),[\alpha /N]+1;1,i\right),\;\mathrm{if}\;\xi =\left(i-1\right)N+1. \end{array}\right.$$

Expression for the mean square of the forecast error is written as:

(11) $$\begin{array}{c} E\left[\Delta^{2}\left(k,N,i\right)\right]=M\left[S^{2\lambda }\left(\nu \right)\right]-M^{2}\left[S^{\lambda }\left(\nu \right)\right]-\\ -\sum_{\mu =k+1}^{i}\sum_{\lambda =1}^{N}D_{\lambda }\left(\mu \right)\rho^{2}\left(\lambda ,\mu ;1,i\right)\beta_{1\mu }^{\left(\lambda \right)}\left(i\right),\;i=\bar{k+1,I}. \end{array}$$

The operation of predictive control consists of checking whether the estimates of the investigated parameter $\hat{s}\left(i\right),\;i=\bar{k+1,I}$ belong to the interval of admissible values:

(12) $$a\leq \hat{s}\left(i\right)\leq b.$$

If condition (12) is not met, a failure is recorded, and a decision to restore the system is made. The conditions $a\leq \hat{s}\left(i\right)$ or $\hat{s}\left(i\right)\leq b$ can also be the criteria for the quality of the functioning of the control system.

The training of the mathematical model and the extrapolator during system operation is carried out based on the formulas:

(13) $$E_{\left(L+1\right)}\left[S^{n}\left(i\right)\right]=\frac{\sum_{l=1}^{L+1}s_{l}^{n}\left(i\right)}{L+1}=\frac{\sum_{l=1}^{L}s_{l}^{n}\left(i\right)L}{\left(L+1\right)L}+\frac{s_{L+1}^{n}\left(i\right)}{L+1}=\frac{E_{\left(L\right)}\left[S^{n}\left(i\right)\right]L+s_{L+1}^{n}\left(i\right)}{\left(L+1\right)};$$

(14) $$\begin{array}{c} D_{n,(L+1)}\left(\nu \right)=\frac{\sum_{l=1}^{L+1}{\left(p_{\nu ,l}^{\left(n\right)}\right)}^{2}}{L}=\frac{\sum_{l=1}^{L}{\left(p_{\nu ,l}^{\left(n\right)}\right)}^{2}\left(L-1\right)}{L\left(L-1\right)}+\frac{{\left(p_{\nu ,L+1}^{\left(n\right)}\right)}^{2}}{L}=\\ =\frac{D_{n,(L)}\left(\nu \right)+\left(s_{L+1}^{n}\left(\nu \right)-{\hat{s}}_{\left(L\right)}^{\left(n-1,\nu \right)}\left(n,\nu \right)\right)}{L}; \end{array}$$

(15) $$\begin{array}{c} \rho_{\left(L+1\right)}\left(n,\nu ;\lambda ,i\right)=\frac{\sum_{l=1}^{L+1}p_{\nu ,l}^{\left(n\right)}\left(s_{l}^{\lambda }-E_{\left(L+1\right)}\left[S^{\lambda }\left(i\right)\right]\right)}{LD_{n,(L+1)}\left(\nu \right)}=\\ =\frac{\rho_{\left(L\right)}\left(n,\nu ;\lambda ,i\right)\left(L-1\right)+\left(s_{L+1}^{n}\left(\nu \right)-{\hat{s}}_{\left(L\right)}^{\left(n-1,\nu \right)}\left(n,\nu \right)\right)\left(s_{L+1}^{\lambda }\left(i\right)-E_{\left(L+1\right)}\left[S^{\lambda }\left(i\right)\right]\right)}{LD_{n,(L+1)}\left(\nu \right)}. \end{array}$$

where $E_{\left(L\right)}\left[S^{n}\left(i\right)\right],\;D_{n,(L)}\left(\nu \right)$ are estimates of the mathematical expectation and variance of random coefficients based on the existing statistical database ($s_{l}^{n}\left(i\right),\;l=\bar{1,L}$) for the systems of this class; $E_{\left(L\right)}\left[S^{n}\left(i\right)\right],\;D_{n,(L)}\left(\nu \right),\;\rho_{\left(L+1\right)}\left(n,\nu ;\lambda ,i\right)$ are refined parameters of the models (3) and (7) using additional statistical data $s_{L}^{n}\left(i\right)$ on the results of the system’s functioning under study.

The proposed extrapolation method was tested on the model of random sequence:

(16) $$S\left(i+1\right)=\frac{5S\left(i\right)}{1+S^{2}\left(i\right)}-0.5S\left(i\right)-0.5S\left(i-1\right)+0.5S\left(i-2\right)+\xi \left(i+1\right)$$

In the first three sections, random sequences are uniformly distributed on the segment [−1;1] and ξ(i) is a uniformly distributed random variable on the segment [−0.1; 0.1].

**Figure 10:**
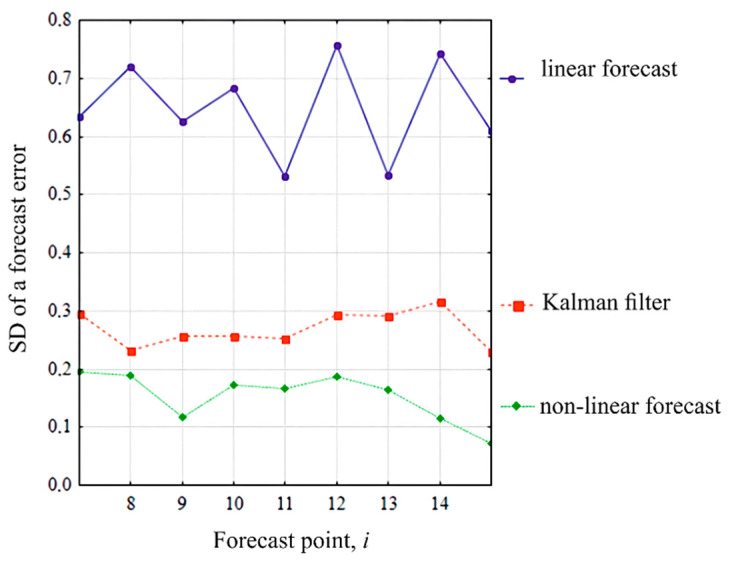
Forecast error standard deviation of the random sequence realizations for various extrapolation algorithms: comparative results.

By the results of 100 extrapolation experiments based on the linear algorithm, the Kalman filter of the order 4 and proposed nonlinear algorithm (of the order 4 based on all previous values $s\left(\nu \right),\;\nu =\bar{1,i}$), the estimates of the standard deviation (SD) were obtained (Figure 10).

Analysis of the standard deviation of the forecast error (Figure 10) indicates a high forecast accuracy when using the nonlinear methods (5) and (6) (curve “non-linear forecast” in Figure 10), in which the stochastic properties of the investigated random sequences are taken into account as much as possible (nonlinearity, use of the full amount of a posteriori information, non-stationarity). The extrapolation accuracy is 3–3.4 times higher compared to the Wiener–Hopf method [126] (“linear forecast” curve in Figure 10) due to the use of non-linear relationships, and 1.5–2.4 times higher compared to the Kalman method [126] due to the use of a larger volume of a posteriori information.

**Figure 11:**
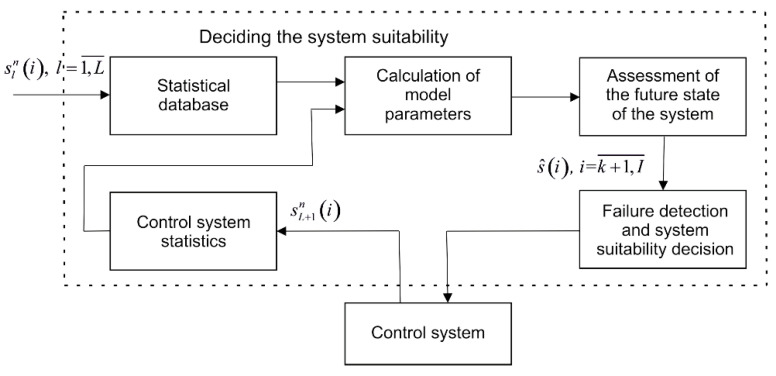
Diagram of the predictive control module functioning.

The diagram in Figure 11 reflects the features of the functioning of the predictive control module.

At the time of putting the system into operation, the estimates $\hat{s}\left(i\right),\;i=\bar{k+1,I}$ of future values are calculated using the Equations (5), (6), (9), and (10), and only based on information about the functioning of systems of this class. In further operations, the machine learning of the model and the extrapolator is performed based on statistical data about the system under study using the Equations (13)–(15).

The module can function in real-time, considering that the initial parameters (5), (6), (9) and (10) of the forecast algorithm can be calculated in advance before the start of the system operation, and the formulae for training (13)–(15) and extrapolation (7) and (8) are computationally simple.

A significant advantage of the proposed method for assessing the operability of a control system is the prevention of failures and, as a result, ensuring its continued operation in future moments. The advantage of the method is also taking into account the individual characteristics of the control system: the accumulation of a priori information about the studied system in the process of operation assessment of operability based on current measurements of the state of the system in real-time. The forecasting used an algorithm that, unlike the known methods (Wiener–Hopf method, Kolmogorov polynomial, Kalman filter, etc.), does not impose any restrictions on the random sequence of changes in system parameters (linearity, monotonicity, ergodicity, stationarity, Markov properties, etc.), which makes it possible to achieve the maximum accuracy of solving the problem of predictive operability monitoring. Patent of Ukraine (Patent No. 73855) defends, and the engineering solution of the considered method is for prediction of the object’s technical state.

## 6. Control System Design and Robot Arm Simulation

The development of an effective control system is a prerequisite for quality machine learning, accurate object recognition, and image classification. Machine learning and intelligent control methods are an important part of designing a control system and arm modeling. It can be used for sorting and orienting objects on the conveyor, remote control of dangerous and/or harmful objects, automatic recognition of objects based on trained models using machine learning technologies, and computer vision [24,27,42,105].

**Figure 12:**
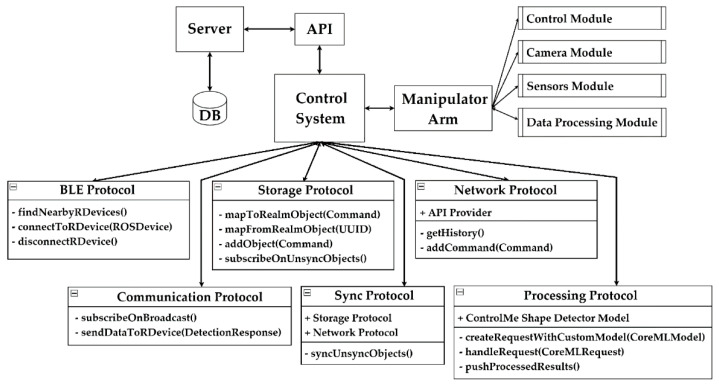
An extended structural diagram of the control system.

In general, the weak sides of analog products are forced to carry out software implementation [1,127]. Before software implementation, it is necessary to design a control system and simulate a manipulator’s arm. Figure 12 shows an illustrative structural diagram of the control system. It shows a list of functions and properties of these services of the control system. These services will not be assigned to the management system itself and will be used only in the right places within the system. For example, the network service may be needed when viewing the log of all actions performed by the robot. Let us consider in more detail the relevant components.

### 6.1. “Control System” Component

General functions can be presented as: communication with a robotic device using a Bluetooth connection; selection of the system operation mode (testing the model, working with device); intelligent device control using a trained neural network; storing information about the state of the device and the current progress of the task; synchronization of intermediate data with the server upon completion of the task by the device; and communication with the server using the REST (Representational State Transfer) API (Application Programming Interface). The list of internal dependencies and additional services consists of a BLE (Bluetooth Low Energy) service for connecting with a robotic device; a service for saving local data; a network service to interact with the server; a communication service to interact with a robotic device; and a data processing service with an artificial intelligence module. The artificial intelligence module is presented as a neural network for pattern recognition and classification. The architecture of this neural network is a convolutional neural network with the YOLOv2 (You Only Look Once) structure. It includes sequential convolution layers with the function ReLU (Rectified Linear Unit), layers pooling for feature map definition, and a fully connected neural network for classification [127,128,129,130].

### 6.2. “Server” Component

General functions are communication with the control system using the REST API, saving history about all devices’ operating states, and intermediate data. The list of internal dependencies are “Fluent ORM” for converting custom queries in the Swift programming language into raw SQL (Structured Query Language); “SQLite” is a relational DBMS (Database Management System) for data storage; and REST API with commands (GET, POST) [131].

### 6.3. “Manipulator Arm” Component

General functions are communication with the control system using a Bluetooth connection; recording an image of the environment using a built-in camera and transmitting the image to the system; getting coordinates of the object from the system; and transforming the coordinates obtained from the system into coordinates of the environment. The “Manipulator arm” component is presented as a simulated manipulator arm using the MoveIt software and the ROS (Robot Operating System) real-time operating system. It has five degrees of freedom and a gripper at the end [132,133,134,135].

**Figure 13:**
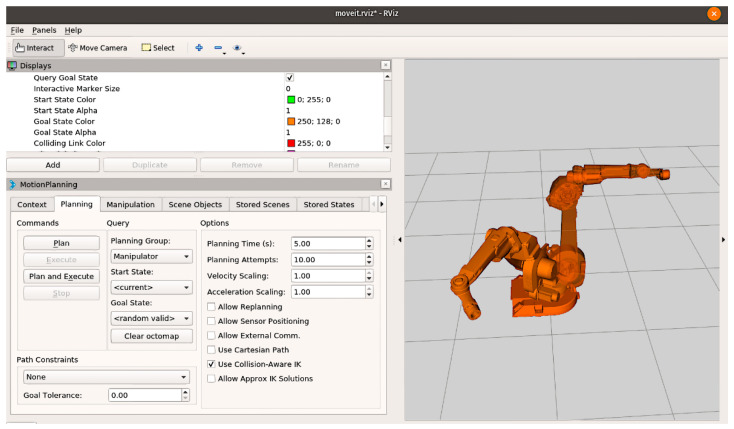
Motion planning with MoveIt software.

MoveIt (Figure 13) is an open-source manipulation software developed by Willow Garageby, Ioan A. Sucan, and Sachin Chitta [133,134,135]. The software offers solutions for mobile manipulation problems, such as kinematics, planning and motion control, 3D perception, and navigation. The MoveIt library is part of the ROS package and is widely used in robotic systems. MoveIt is great for developers because it can be easily customized for any job [133,134,135].

For designing a control system using MoveIt software, it is necessary to acquire the URDF (Universal Robot Description Format) file of the device (in our case, it is the URDF of the manipulator’s arm). This file contains elemental arm composition, length, and joints. MoveIt comes with MoveIt Assistant that helps to configure and simplify things to a great extent [133]. Based on the batch files with the robot configurations and the MoveIt Rviz (Figure 13) configuration environment, it is possible to perform operations (movement planning, manipulation, and others) on a robotic device [133,134,135].

The novelty of the obtained results is (a) the development of the original structure of the control system with the implementation of interaction protocols of system components and the integration of an artificial intelligence module for the recognition and classification of objects for manipulator arm missions; and (b) modeling of the developed control system in the MoveIt environment with adjustment of the corresponding parameters for different manipulator arm missions.

## 7. Object Recognition in Robot Working Space Using Convolutional Neural Network

**Figure 14:**
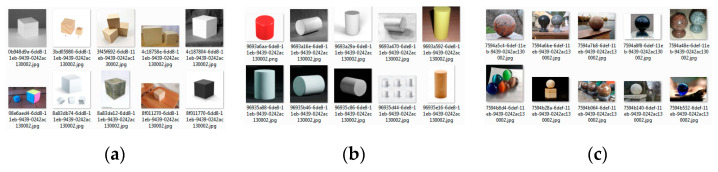
Fragments of the datasets for: (**a**) cube; (**b**) cylinder; (**c**) sphere.

When designing an intelligent control module, it is necessary to find sets of images that will be used to perform the neural network’s learning process. Intelligent control is carried out by one of the methods of machine learning, namely pattern recognition and classification, using artificial neural networks [1,3,24,25,26,27,42,99]. The robotic device sends the query to gather an image from the environment, the control system processes the obtained image and sends the processing results to the robotic device. The result of this processing is the fact that the object belongs to one of the classes and its coordinates, relative to the resulting image. In our case, it is necessary to find three datasets of images for each class: cube, cylinder, sphere (partial image datasets in Figure 14).

Images with several classes were also found, for example, with three cubes and two cylinders. In this case, images with one class could be with one or more objects, such as one or five spheres. The resulting total dataset contains 213 images, including 70 images of the class “Cube”, 70 images of the class “Cylinder”, 71 images of the class “Sphere”, and 2 images with several different classes [99].

**Figure 15:**
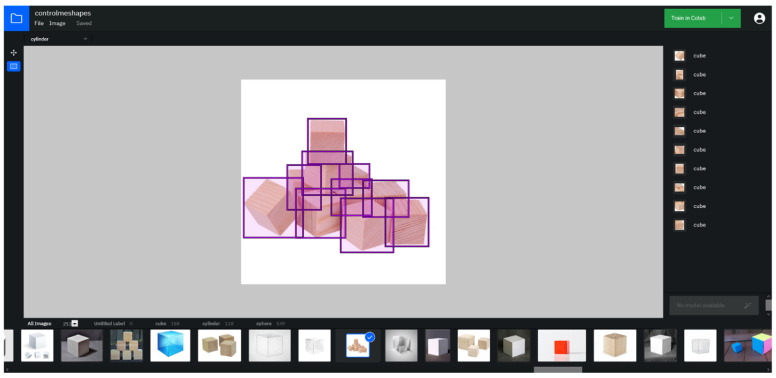
The process of adding annotations to images.

Since the training of neural networks for such a class of tasks takes place with the teacher, it is necessary to add annotations for the resulting total dataset, including the coordinates of the object belonging to the class. Using the resource https://cloud.annotations.ai/ (accessed on 8 February 2021), authors create a project, transfer the prepared sets of images, and add annotations for each class. Figure 15 shows the process of adding annotations to images.

Figure 15 shows that one image contains 10 objects of the same class “Cube”, in our case, 10 annotations were added for 10 cubes. Annotations are a rectangle where there is an object of a certain class, with coordinates (X, Y, width, height). After adding annotations for all data, the export of the data is prepared in the format of Create ML [99,128].

**Figure 16:**
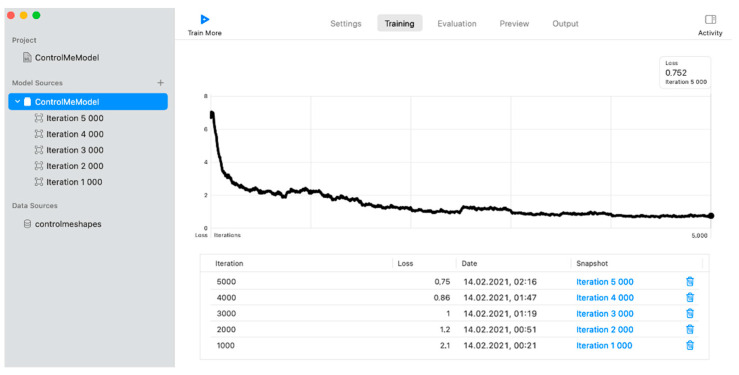
The training process in Create ML.

The paper uses a convolutional neural network with YOLOv2 architecture. To train this type of network, a set of images and an annotation in .json format is necessary, with detailed information about the location of the class object (coordinates in 2D space) and the class itself. The following parameters were chosen for the neural network using Create ML [128,129,130,136] (Figure 16): the algorithm is the complete network (trains a complete object detection network based on YOLOv2 architecture); the number of epochs is 5000; the batch size is automatic; and the grid size is 13 × 13.

Additional information about learning (training) outcomes is presented in Table 1.

The neural network model has achieved a training accuracy of 100% in 5000 epochs. The model was created, trained, and tested on a 2016 MacBook Air with a 1.6 GHz i5 CPU.

**Figure 17:**
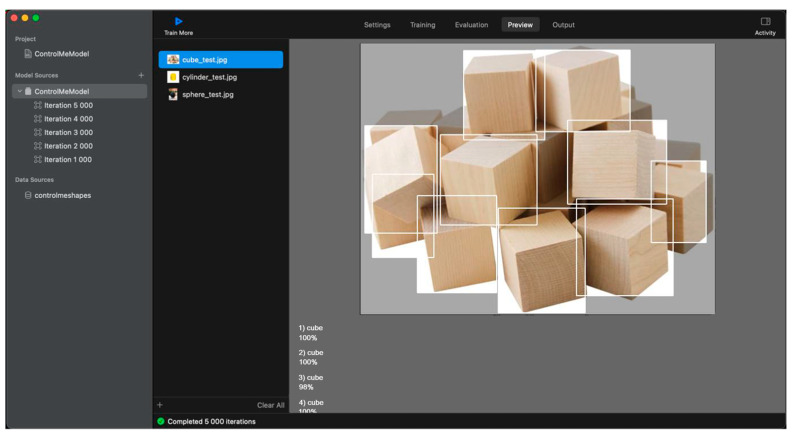
Testing (recognition) results of the object class “Cube”.

To check the results of training the neural network, we prepared a set of images that contain objects belonging to the specified classes and were not in the set of images for training. The testing (recognition) results (Figure 17) show that the objects (10 cubes) in the image were recognized with 99.8% accuracy (accuracy of recognition of one of the cubes was 98%, the others were 100%). However, it should be noted that in some cases, when the image is distorted, the recognition accuracy is reduced. A total of 50 images with different classes and number of objects were tested, and the overall accuracy was 99%.

In the developed software implementation, the communication between the client and the server is based on the HTTP (HyperText Transfer Protocol) protocol. With the help of HTTP requests (GET, POST, PUT, DELETE), communication takes place. The client sends a request to one of the available endpoints, the server accepts the request, processes the data, and returns a response [99].

To expand the capabilities of the intelligent control system, the authors have increased the number of classes for recognition from three (“Cube”, “Sphere”, “Cylinder”) to five (“Cube”, “Sphere”, “Cylinder”, “Cone”, and “Pyramid”). Now, the resulting total dataset contains 225 images, including 52 images of the class “Sphere”, 45 images of the class “Cone”, 44 images of the class “Cube”, 42 images of the class “Pyramid”, and 42 images of the class “Cylinder”. The input images had a resolution of 416 by 416 pixels.

Additional information about learning (training) outcomes for five classes of recognition is presented in Table 2.

In this case, the model was created, trained, and tested on a more powerful 2017 MacBook Air with a 1.8 GHz i5 CPU. The neural network model achieved a training accuracy of 100% in 5000 epochs with less loss and time according to data in Table 1.

**Figure 18:**
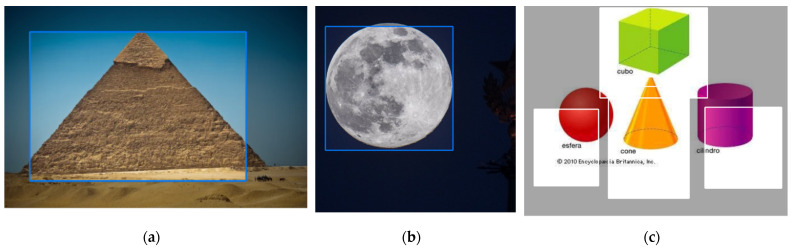
Testing (recognition) results: (**a**) class “Pyramid”; (**b**) class “Sphere”; (**c**) several objects of different classes.

In addition, the accuracy of recognition (classification) is very high. For example, the same objects in different numbers that belong to the same class, the network recognizes with 100% accuracy (Figure 18a,b). Recognition accuracy decreases if several objects of different classes are in the same image (Figure 18c). For example, the “Cylinder” object (Figure 18c) belongs to the following classes with varying accuracy: the class “Cylinder” with 91%, the class “Sphere” with 4%, the class “Cube” with 3%, the classes “Pyramid” and “Cone” with 1%. A similar situation with the “Cone” object refers to the following classes with varying accuracy: the class “Cone” with 97%, and the class “Pyramid” with 3%. However, still displays a high accuracy of recognition (classification).

A similar dataset (https://data.wielgosz.info, accessed on 10 Decemebr 2021) with five geometric figures (cone, cube, cylinder, sphere, torus) was chosen to study the influence of sample size, neural network architecture, and learning parameters on recognition accuracy. The dataset has training and test samples. The size of the training sample is 40,000 images (8000 images of each figure). The size of the testing sample is 10,000 images (2000 images of each object).

Different transformations were applied to each sample. Both training and test images were normalized and cropped to 224 by 224 pixels. In addition, for greater accuracy, additional transformations were applied to the training sample, including random rotations and shifts of the image center.

The authors chose the ResNet34 architecture. The Torch library for Python was used to work with it. The neural network trained at Google Colab was on the GPU [26,27]. The ResNet34 model was downloaded for training, and the number of outputs was changed from 1000 (default) to 5 (each output is a figure class). In the third epoch, the testing accuracy decreased (overfitting/overtraining took place), so the process was stopped. The best result in the second epoch was 98%. Additional information about training and testing outcomes for five classes for recognition is presented in Table 3.

Using fine-tuning technology, the authors increased the testing accuracy to 99.2%. Additional information about training and testing outcomes for five classes of recognition using fine-tuning technology is presented in Table 4.

The results (Table 4) show that after the third epoch there is overtraining. So, let us focus on the third epoch: the training accuracy is 99.75% and the testing accuracy is 99.2%. The obtained numerical results, compared to other similar studies [68] in which a K-nearest neighbor algorithm and a support vector machine algorithm were used for recognition and classification, in this case, the values of accuracy were 84.2% and 81.6%, respectively. This proves the advantage of neural networks in such problems.

The authors also investigated the effect of dataset size on recognition accuracy. For the study, the number of images of each class in the training and test datasets was reduced to 200 images. Thus, 1000 training images and 1000 test images remained. The results of the use of the neural network with ResNet34 architecture and fine-tuning technology are presented in Table 5.

The results (Table 5) show that the testing accuracy gradually increases from 88.4% to 98%. However, the neural network trains more slowly than on a full dataset. The sample size has a significant impact on the quality of training and the number of epochs.

The Vapor web framework was chosen to implement the server part. According to the general list of server functions, communication with the client part is necessary. This can be performed using the HTTP protocol. Vapor Toolbox is required when working with Vapor. To download and install this set, run the following command (brew tap vapor/tap && brew install vapor/tap/vapor) in the Terminal window. After executing this command, access to the Vapor Toolbox appears [137].

Use the Postman program to test the developed application interface. Postman allows verifying requests from the client to the server and the server’s response to requests. Then create a request to add a command to the scheme. Select the POST method, specify the endpoint address and port, form the body of the query, which will specify the attributes of the model, and click send. Then create a request to receive all commands from the scheme. Select the GET method, specify the endpoint address and port, click send. You can see the successful query code and the list of received items [131].

With the help of the abstract network layer Moya, we can configure communication with the server. To perform this, specify the server address, endpoints, HTTP method, request type, and headers [126].

**Figure 19:**
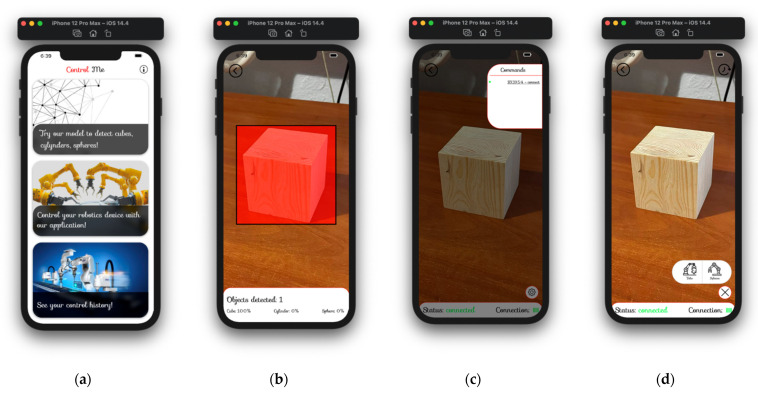
Device control system: (**a**) Module “Dashboard”; (**b**) Module “Testing”; (**c**) Module “Control”: submodule “Device connection”; (**d**) Module “Control”: submodule “Capture by device”.

The device control system (Figure 19) can be divided into the following modules: module “Dashboard”, module “Testing”, and module “Control” [99].

The “Dashboard” module (Figure 19a) is presented as a list of possible options for using the system: testing a trained model using a camera of a mobile device (imitation of a manipulator camera), connecting to a robotic device and control, and viewing the history of actions on devices. The “Testing” module (Figure 19b) is used to test a trained convolutional network with the YOLOv2 architecture and the ReLU activation function in the convolution layers. This module simulates the transmission of a video stream from a robotic device and performs image processing using the specified network. The processing results are indicated in the block containing the number of objects recognized in the current image and belonging to the specified class. Module “Control” (Figure 19c,d) is the main unit of this intelligent control system. It contains the following services: local data storage service, network service, communication service, synchronization service, and data processing service. All these services are realized using the Swinject container. Module “Control”: submodule “Device connection” (Figure 19c) is needed to search for robotic devices that are in range and connect to them using Bluetooth technology. After a successful connection, a command is issued, which contains communication protocols with the device. Module “Control”: submodule “Capture by device” (Figure 19d) is designed to directly capture the recognized object and control it.

The “History” module is presented as a collection with objects obtained using a network service, which in turn is realized using a Swinject container. The collection header contains a drop-down list with possible sorting options (by device ID, context, timestamp).

The novelty of the obtained results consist of (a) expanding capabilities of the intelligent control system by increasing the number of recognition classes to five classes (pyramid, cone, cube, cylinder, and sphere) which, in contrast to the existing model with three recognition classes (cube, cylinder, and sphere), has a wider area of object recognition for further capture by the manipulator; (b) the neural network model with the ResNet34 architecture gained further development through the complex application of optimization techniques, including random rotations and shifts of the image center, splitting the training sample into batches with 64 images, using fine-tuning technology, which, in contrast to the existing model, provides increasing the training accuracy by 1.73% (from 98.02% to 99.75%) and the testing accuracy by 1.2% (from 98.0% to 99.2%).

## 8. Conclusions

This article presents an analytical review of the machine learning methods applied in different areas of human activity with a focus on robotic systems. Special attention is paid to increasing the efficiency of the sensor and control information processing in the advanced multi-component robotic complexes that function in non-stationary, uncertain, or unknown working environments. MCRC consists of a moving mobile robot and an adaptive robot with a fixed base (manipulator with adaptive gripper) as well as the sensor and control systems.

Authors’ contributions demonstrate the increasing control quality and extending functioning properties of MCRC’s robotic components by using (a) a fuzzy logic approach for recognition of the slippage direction of a manipulated object in the robot fingers in collision with an obstacle; (b) neuro and neuro-fuzzy approaches for the design of intelligent controllers and clamping force observers of mobile robots with magnetically controlled wheels which can move working tools or an adaptive robot with the fixed base on inclined or ceiling ferromagnetic surfaces (ship hull, etc.); (c) a canonical decomposition approach from statistical learning theory for the prediction of robot control system states during robot mission in the nonstationary environment.

To improve the reliability of control systems, it is proposed to use an operability-monitoring module that allows for the determination of possible system failures at future points in time. The used algorithm for forecast parameters of control systems, unlike the known methods (Wiener–Hopf method, Kolmogorov polynomial, Kalman filter, etc.), does not impose any restrictions on the random sequence of changes in system parameters (linearity, monotonicity, ergodicity, stationarity, Markov properties, etc.), which makes it possible to achieve the maximum accuracy of solving the problem of predictive operability monitoring. The results of the numerical experiment confirmed its high efficiency (the relative extrapolation error is 2–3%).

Besides, the authors illustrate the efficiency of software used for the design of robot control systems and training of the developed convolutional neural network for recognition of the objects from different classes based on video-sensor information processing.

A general structural diagram of the control system is formed at the system design stage, an entity-table with a relational DBMS is presented and a robotic arm is modeled using MoveIt software. Upon completion of the design, the stage of software implementation of the control system was carried out. The server is implemented using the Vapor web framework, the control system—using the Swift programming language and other technologies, and the configuration and creation of a neural network—using the CreateML framework.

A neural network with the ResNet34 architecture trains quickly (3 epochs to achieve 99.2% testing accuracy) in comparison with Create ML (YOLOv2 architecture). The results of the authors’ investigation of the impact of dataset size on the training accuracy and testing accuracy are: (a) the training accuracy gradually increased from the 1st to the 5th epoch (77.8%, 94.8%, 95.3%, 96.7%, and 99.1%, respectively); (b) the testing accuracy increased from the 1st (88.4%) to the 5th epoch (98.0%); and (c) the neural network trained slower (122 s in 5 epochs) on the small size dataset (1000 training images) compared with the full dataset (75 s in 3 epochs), with 40,000 training images.

Fine-tuning technology with ResNet34 architecture increases training accuracy (to 99.75%) and testing accuracy (to 99.2%) for recognition of five different classes using the Torch library (Python).

## 9. Patents

Patent No. 52080, Ukraine, 2010. Y. P. Kondratenko, et al. Intelligent sensor system.

Patent No. 45369, Ukraine, 2009. Y. P. Kondratenko, et al. Propulsion wheel of mobile robot.

Patent No. 47369, Ukraine, 2010. Y. P. Kondratenko, et al. Method of magnetically operated displacement of mobile robot.

Patent No. 100341, Ukraine, 2015. V. O. Kushnir, Y. P. Kondratenko, et al. Mobile robot for mechanical clearing ship hull.

Patent No. 73855, Ukraine, 2012. I. P. Atamanyuk, Y. P. Kondratenko. Method for prediction of object technical state.

## Figures and Tables

**Table 1:** Training outcomes using Create ML.

Number of Epochs	Loss	Time in Seconds	Training Accuracy in %	Validation Accuracy in %
1000	2.123	1860	87	82
2000	1.211	3660	95	90
3000	1.044	5340	95	93
4000	0.857	7020	99	93
5000	0.752	8760	100	95

**Table 2:** Training outcomes for recognition of five classes using Create ML.

Number of Epochs	Loss	Time in Seconds	Training Accuracy in %	Validation Accuracy in %
450	1.7	780	85	80
510	1.7	900	86	81
1000	1.2	1800	87	82
2000	0.95	3540	95	90
3000	0.73	5220	95	93
4000	0.68	6960	99	96
5000	0.63	8700	100	100

**Table 3:** Training and testing outcomes for recognition of 5 classes by Torch library (Python).

Number of Epochs	Training Loss	Testing Loss	Training Accuracy in %	Testing Accuracy in %
1	0.1187	0.0857	96.21	97.07
2	0.0599	0.0611	98.02	98.00
3	0.0515	0.0661	98.28	97.87

**Table 4:** Training and testing outcomes for recognition of 5 classes using fine-tuning technology by the Torch library (Python).

Number of Epochs	Training Loss	Testing Loss	Training Accuracy in %	Testing Accuracy in %
1	0.0469	0.0341	98.38	98.68
2	0.0092	0.0582	99.67	97.68
3	0.0060	0.0263	99.75	99.20
4	0.0058	0.0677	99.76	97.16

**Table 5:** Training and testing outcomes for recognition of five classes (1000 training images and 1000 test images) using fine-tuning technology by the Torch library (Python).

Number of Epochs	Training Loss	Testing Loss	Training Accuracy in %	Testing Accuracy in %
1	0.6128	0.3412	77.80	88.40
2	0.1623	0.1902	94.80	93.60
3	0.1330	0.2070	95.30	90.90
4	0.1011	0.1240	96.70	96.80
5	0.0302	0.0601	99.10	98.00

## Data Availability

Used dataset with geometric figures (https://data.wielgosz.info/, accessed on 10 Decemebr 2021).

## References

[1] Kawana E., Yasunobu S. (2007). An Intelligent Control System Using Object Model by Real-Time Learning. Proceedings of the SICE Annual Conference.

[2] Ayvaz S., Alpay K. (2021). Predictive maintenance system for production lines in manufacturing: A machine learning approach using IoT data in real-time. Expert Syst. Appl..

[3] Kondratenko Y., Duro R. (2015). Advances in Intelligent Robotics and Collaborative Automation.

[4] Kondratenko Y., Khalaf P., Richter H., Simon D. (2019). Fuzzy Real-Time Multi-objective Optimization of a Prosthesis Test Robot Control System. Advanced Control Techniques in Complex Engineering Systems: Theory and Applications, Dedicated to Professor Vsevolod M. Kuntsevich. Studies in Systems, Decision and Control.

[5] Guo J., Xian B., Wang F., Zhang X. (2013). Development of a three degree-of-freedom testbed for an unmanned helicopter and attitude control design. Proceedings of the 32nd Chinese Control Conference.

[6] Kondratenko Y., Klymenko L., Kondratenko V., Kondratenko G., Shvets E. (2013). Slip Displacement Sensors for Intelligent Robots: Solutions and Models. Proceedings of the 2013 IEEE 7th International Conference on Intelligent Data Acquisition and Advanced Computing Systems (IDAACS).

[7] Derkach M., Matiuk D., Skarga-Bandurova I. (2020). Obstacle Avoidance Algorithm for Small Autonomous Mobile Robot Equipped with Ultrasonic Sensors. Proceedings of the 2020 IEEE 11th International Conference on Dependable Systems, Services and Technologies (DESSERT).

[8] Tkachenko A.N., Brovinskaya N.M., Kondratenko Y.P. (1983). Evolutionary adaptation of control processes in robots operating in non-stationary environments. Mech. Mach. Theory.

[9] Kondratenko Y., Khademi G., Azimi V., Ebeigbe D., Abdelhady M., Fakoorian S.A., Barto T., Roshanineshat A., Atamanyuk I., Simon D. (2016). Robotics and Prosthetics at Cleveland State University: Modern Information, Communication, and Modeling Technologies. Communications in Computer and Information Science, Proceedings of the 12th International Conference on Information and Communication Technologies in Education, Research, and Industrial Applications (ICTERI 2016), Kyiv, Ukraine, 21–24 June 2016.

[10] Patel U., Hatay E., D’Arcy M., Zand G., Fazli P. A Collaborative Autonomous Mobile Service Robot. Proceedings of the AAAI Fall Symposium on Artificial Intelligence for Human-Robot Interaction (AI-HRI).

[11] Li Z., Huang Z. (2013). Design of a type of cleaning robot with ultrasonic. J. Theor. Appl. Inf. Technol..

[12] Kondratenko Y.P., Gil-Lafuente A.M., Zopounidis C. (2015). Robotics, Automation and Information Systems: Future Perspectives and Correlation with Culture, Sport and Life Science. Decision Making and Knowledge Decision Support Systems. Lecture Notes in Economics and Mathematical Systems.

[13] Taranov M.O., Kondratenko Y. (2018). Models of Robot’s Wheel-Mover Behavior on Ferromagnetic Surfaces. Int. J. Comput..

[14] D’Arcy M., Fazli P., Simon D. (2017). Safe Navigation in Dynamic, Unknown, Continuous, and Cluttered Environments. Proceedings of the IEEE International Symposium on Safety, Security, and Rescue Robotics (SSRR).

[15] Taranov M., Wolf C., Rudolph J., Kondratenko Y. (2017). Simulation of Robot’s Wheel-Mover on Ferromagnetic Surfaces. Proceedings of the 9th IEEE International Conference on Intelligent Data Acquisition and Advanced Computing Systems: Technology and Applications (IDAACS).

[16] Driankov D., Saffiotti A. (2013). Fuzzy Logic Techniques for Autonomous Vehicle Navigation.

[17] Kondratenko Y.P., Rudolph J., Kozlov O.V., Zaporozhets Y.M., Gerasin O.S. (2017). Neuro-fuzzy observers of clamping force for magnetically operated movers of mobile robots. Tech. Electrodyn..

[18] Gerasin O.S., Topalov A.M., Taranov M.O., Kozlov O.V., Kondratenko Y.P. (2020). Remote IoT-based control system of the mobile caterpillar robot. Proceedings of the 16th International Conference on ICT in Education, Research and Industrial Applications. Integration, Harmonization and Knowledge Transfer (ICTERI 2020).

[19] Inoue K., Kaizu Y., Igarashi S., Imou K. (2019). The development of autonomous navigation and obstacle avoidance for a robotic mower using machine vision technique. IFAC PapersOnLine.

[20] Zhang Y., Zhao J., Sun J. (2020). Robot Path Planning Method Based on Deep Reinforcement Learning. Proceedings of the 2020 IEEE 3rd International Conference on Computer and Communication Engineering Technology (CCET).

[21] Qijie Z., Yue Z., Shihui L. (2020). A path planning algorithm based on RRT and SARSA (λ) in unknown and complex conditions. Proceedings of the 2020 Chinese Control and Decision Conference (CCDC).

[22] Koza J.R., Bennett F.H., Andre D., Keane M.A., Gero J.S., Sudweeks F. (1996). Automated Design of Both the Topology and Sizing of Analog Electrical Circuits Using Genetic Programming. Artificial Intelligence in Design ’96.

[23] Hu J., Niu H., Carrasco J., Lennox B., Arvin F. (2020). Voronoi-Based Multi-Robot Autonomous Exploration in Unknown Environments via Deep Reinforcement Learning. IEEE Trans. Veh. Technol..

[24] Sokoliuk A., Kondratenko G., Sidenko I., Kondratenko Y., Khomchenko A., Atamanyuk I. (2020). Machine Learning Algorithms for Binary Classification of Liver Disease. Proceedings of the 2020 IEEE International Conference on Problems of Infocommunications. Science and Technology (PIC S&T).

[25] Sheremet A., Kondratenko Y., Sidenko I., Kondratenko G. (2021). Diagnosis of Lung Disease Based on Medical Images Using Artificial Neural Networks. Proceedings of the 2021 IEEE 3rd Conference on Electrical and Computer Engineering (UKRCON).

[26] Kondratenko Y., Sidenko I., Kondratenko G., Petrovych V., Taranov M., Sova I. (2020). Artificial Neural Networks for Recognition of Brain Tumors on MRI Images. Communications in Computer and Information Science, Proceedings of the 16th International Conference on Information and Communication Technologies in Education, Research, and Industrial Applications (ICTERI 2020), Kharkiv, Ukraine, 6–10 October 2020.

[27] Sova I., Sidenko I., Kondratenko Y. (2020). Machine learning technology for neoplasm segmentation on brain MRI scans. Proceedings of the 2020 PhD Symposium at ICT in Education, Research, and Industrial Applications (ICTERI-PhD 2020).

[28] Kassahun Y., Yu B., Tibebu A.T., Stoyanov D., Giannarou S., Metzen J.H., Poorten E.V. (2016). Surgical robotics beyond enhanced dexterity instrumentation: A survey of machine learning techniques and their role in intelligent and autonomous surgical actions. Int. J. Comput. Assist. Radiol. Surg..

[29] Striuk O., Kondratenko Y. (2021). Generative Adversarial Neural Networks and Deep Learning: Successful Cases and Advanced Approaches. Int. J. Comput..

[30] Mohammadi A., Meniailov A., Bazilevych K., Yakovlev S., Chumachenko D. (2021). Comparative study of linear regression and sir models of COVID-19 propagation in Ukraine before vaccination. Radioelectron. Comput. Syst..

[31] Striuk O., Kondratenko Y., Sidenko I., Vorobyova A. (2020). Generative adversarial neural network for creating photorealistic images. Proceedings of the 2nd IEEE International Conference on Advanced Trends in Information Theory (ATIT).

[32] Ren C., Kim D.-K., Jeong D. (2020). A Survey of Deep Learning in Agriculture: Techniques and Their Applications. J. Inf. Process. Syst..

[33] Kumar M., Kumar A., Palaparthy V.S. (2021). Soil Sensors-Based Prediction System for Plant Diseases Using Exploratory Data Analysis and Machine Learning. IEEE Sens. J..

[34] Atamanyuk I., Kondratenko Y., Sirenko N., Berger-Vachon C., Gil Lafuente A., Kacprzyk J., Kondratenko Y., Merigó J., Morabito C. (2018). Management System for Agricultural Enterprise on the Basis of Its Economic State Forecasting. Complex Systems: Solutions and Challenges in Economics, Management and Engineering. Studies in Systems, Decision and Control.

[35] Atamanyuk I., Kondratenko Y., Poltorak A., Sirenko N., Shebanin V., Baryshevska I., Atamaniuk V., Ermolaev V., Mallet F., Yakovyna V., Kharchenko V., Kobets V., Kornilowicz A., Kravtsov H., Nikitchenko M., Semerikov S., Spivakovsky A. (2019). Forecasting of Cereal Crop Harvest on the Basis of an Extrapolation Canonical Model of a Vector Random Sequence. Proceedings of the 15th International Conference on Information and Communication Technologies in Education, Re-search, and Industrial Applications. Volume II: Workshops (ICTERI 2019).

[36] Atamanyuk I.P., Kondratenko Y.P., Sirenko N.N. (2016). Forecasting Economic Indices of Agricultural Enterprises Based on Vector Polynomial Canonical Expansion of Random Sequences. Proceedings of the 12th International Conference on Information and Communication Technologies in Education, Research, and Industrial Applications (ICTERI 2016).

[37] Werners B., Kondratenko Y., Berger-Vachon C., Gil Lafuente A., Kacprzyk J., Kondratenko Y., Merigó J., Morabito C. (2018). Alternative Fuzzy Approaches for Efficiently Solving the Capacitated Vehicle Routing Problem in Conditions of Uncertain Demands. Complex Systems: Solutions and Challenges in Economics, Management and Engineering. Studies in Systems, Decision and Control.

[38] Kondratenko G.V., Kondratenko Y.P., Romanov D.O. Fuzzy Models for Capacitive Vehicle Routing Problem in Uncertainty. Proceedings of the 17th International DAAAM Symposium Intelligent Manufacturing and Automation: Focus on Mechatronics & Robotics.

[39] Zinchenko V., Kondratenko G., Sidenko I., Kondratenko Y. (2020). Computer vision in control and optimization of road traffic. Proceedings of the 2020 IEEE 3rd International Conference on Data Stream Mining and Processing (DSMP).

[40] Jingyao W., Manas R.P., Nallappan G. (2022). Machine learning-based human-robot interaction in ITS. Inf. Process. Manag..

[41] Leizerovych R., Kondratenko G., Sidenko I., Kondratenko Y. (2020). IoT-complex for Monitoring and Analysis of Motor Highway Condition Using Artificial Neural Networks. Proceedings of the 2020 IEEE 11th International Conference on Dependable Systems, Services and Technologies (DESSERT).

[42] Kondratenko Y.P., Kondratenko N.Y., Casey A. (2016). Reduced library of the soft computing analytic models for arithmetic operations with asymmetrical fuzzy numbers. Soft Computing: Developments, Methods and Applications. Series: Computer Science, Technology and Applications.

[43] Gozhyj A., Nechakhin V., Kalinina I. (2020). Solar Power Control System based on Machine Learning Methods. Proceedings of the 2020 IEEE 15th International Conference on Computer Sciences and Information Technologies (CSIT).

[44] Chornovol O., Kondratenko G., Sidenko I., Kondratenko Y. Intelligent forecasting system for NPP’s energy production. Proceedings of the 2020 IEEE 3rd International Conference on Data Stream Mining and Processing (DSMP).

[45] Borysenko V., Kondratenko G., Sidenko I., Kondratenko Y. (2020). Intelligent forecasting in multi-criteria decision-making. Proceedings of the 3rd International Workshop on Computer Modeling and Intelligent Systems (CMIS-2020).

[46] Lavrynenko S., Kondratenko G., Sidenko I., Kondratenko Y. (2020). Fuzzy Logic Approach for Evaluating the Effectiveness of Investment Projects. Proceedings of the 2020 IEEE 15th International Scientific and Technical Conference on Computer Sciences and Information Technologies (CSIT).

[47] Bidyuk P.I., Gozhyj A., Kalinina I., Vysotska V., Vasilev M., Malets M. (2020). Forecasting nonlinear nonstationary processes in machine learning task. Proceedings of the 2020 IEEE 3rd International Conference on Data Stream Mining and Processing (DSMP).

[48] Osborne M.A., Roberts S.J., Rogers A., Ramchurn S.D., Jennings N.R. (2008). Towards Real-Time Information Processing of Sensor Network Data Using Computationally Efficient Multi-output Gaussian Processes. Proceedings of the 2008 International Conference on Information Processing in Sensor Networks (IPSN 2008).

[49] Atamanyuk I., Shebanin V., Kondratenko Y., Havrysh V., Lykhach V., Kramarenko S. (2020). Identification of the Optimal Parameters for Forecasting the State of Technical Objects Based on the Canonical Random Sequence Decomposition. Proceedings of the 2020 IEEE 11th International Conference on Dependable Systems, Services and Technologies (DESSERT).

[50] Li X., Shang W., Cong S. (2020). Model-Based Reinforcement Learning for Robot Control. Proceedings of the 2020 5th International Conference on Advanced Robotics and Mechatronics (ICARM).

[51] Alsamhi S.H., Ma O., Ansari S. (2020). Convergence of Machine Learning and Robotics Communication in Collaborative Assembly: Mobility, Connectivity and Future Perspectives. J. Intell. Robot. Syst..

[52] Samadi G.M., Jond H.B. (2021). Speed Control for Leader-Follower Robot Formation Using Fuzzy System and Supervised Machine Learning. Sensors.

[53] Krishnan S., Wan Z., Bharadwaj K., Whatmough P., Faust A., Neuman S., Wei G.-Y., Brooks D., Reddi V.J. (2021). Machine learning-based automated design space exploration for autonomous aerial robots. arXiv.

[54] El-Shamouty M., Kleeberger K., Lämmle A., Huber M.F. (2019). Simulation-driven machine learning for robotics and automation. Tech. Mess..

[55] Rajawat A.S., Rawat R., Barhanpurkar K., Shaw R.N., Ghosh A. (2021). Robotic process automation with increasing productivity and improving product quality using artificial intelligence and machine learning. Artif. Intell. Future Gener. Robot..

[56] Yaseerz A., Chen H. (2021). Machine learning based layer roughness modeling in robotic additive manufacturing. J. Manuf. Process..

[57] Wang X.V., Pinter J.S., Liu Z., Wang L. (2021). A machine learning-based image processing approach for robotic assembly system. Procedia CIRP.

[58] Mayr A., Kißkalt D., Lomakin A., Graichen K., Franke J. (2021). Towards an intelligent linear winding process through sensor integration and machine learning techniques. Procedia CIRP.

[59] Al-Mousawi A.J. (2020). Magnetic Explosives Detection System (MEDS) based on wireless sensor network and machine learning. Measurement.

[60] Martins P., Sá F., Morgado F., Cunha C. (2020). Using machine learning for cognitive Robotic Process Automation (RPA). Proceedings of the 2020 15th Iberian Conference on Information Systems and Technologies (CISTI).

[61] Segreto T., Teti R. (2019). Machine learning for in-process end-point detection in robot-assisted polishing using multiple sensor monitoring. Int. J. Adv. Manuf. Technol..

[62] Klingspor V., Morik K., Rieger A. (1996). Learning Concepts from Sensor Data of a Mobile Robot. Mach. Learn..

[63] Zheng Y., Song Q., Liu J., Song Q., Yue Q. (2020). Research on motion pattern recognition of exoskeleton robot based on multimodal machine learning model. Neural. Comput. Appl..

[64] Radouan A.M. (2021). Deep Learning for Robotics. J. Data Anal. Inf. Process..

[65] Teng X., Lijun T. (2021). Adoption of Machine Learning Algorithm-Based Intelligent Basketball Training Robot in Athlete Injury Prevention. Front. Neurorobot..

[66] Shih B., Shah D., Li J., Thuruthel T.G., Park J.-L., Iida F., Bao Z., Kramer-Bottiglio R., Tolley M.T. (2020). Electronic skins and machine learning for intelligent soft robots. Sci. Robot..

[67] Ibrahim A., Younes H., Alameh A., Valle M. (2020). Near Sensors Computation based on Embedded Machine Learning for Electronic Skin. Procedia Manuf..

[68] Keser S., Hayber Ş.E. (2021). Fiber optic tactile sensor for surface roughness recognition by machine learning algorithms. Sens. Actuators A.

[69] Gonzalez R., Fiacchini M., Iagnemma K. (2018). Slippage prediction for off-road mobile robots via machine learning regression and proprioceptive sensing. Rob. Auton. Syst..

[70] Wei P., Wang B. (2020). Multi-sensor detection and control network technology based on parallel computing model in robot target detection and recognition. Comput. Commun..

[71] Martinez-Hernandez U., Rubio-Solis A., Prescott T.J. (2020). Learning from sensory predictions for autonomous and adaptive exploration of object shape with a tactile robot. Neurocomputing.

[72] Scholl C., Tobola A., Ludwig K., Zanca D., Eskofier B.M. (2021). A Smart Capacitive Sensor Skin with Embedded Data Quality Indication for Enhanced Safety in Human–Robot Interaction. Sensors.

[73] Joshi S., Kumra S., Sahin F. (2020). Robotic Grasping using Deep Reinforcement Learning. Proceedings of the 2020 IEEE 16th International Conference on Automation Science and Engineering (CASE).

[74] Bilal D.K., Unel M., Tunc L.T., Gonul B. (2022). Development of a vision based pose estimation system for robotic machining and improving its accuracy using LSTM neural networks and sparse regression. Robot. Comput. Integr. Manuf..

[75] Mishra S., Jabin S., Shaw R., Ghosh A., Balas V., Bianchini M. (2021). Recent trends in pedestrian detection for robotic vision using deep learning techniques. Artificial Intelligence for Future Generation Robotics.

[76] Long J., Mou J., Zhang L., Zhang S., Li C. (2021). Attitude data-based deep hybrid learning architecture for intelligent fault diagnosis of multi-joint industrial robots. J. Manuf. Syst..

[77] Subha T.D., Subash T.D., Claudia Jane K.S., Devadharshini D., Francis D.I. (2020). Autonomous Under Water Vehicle Based on Extreme Learning Machine for Sensor Fault Diagnostics. Mater. Today.

[78] Severo de Souza P.S., Rubin F.P., Hohemberger R., Ferreto T.C., Lorenzon A.F., Luizelli M.C., Rossi F.D. (2020). Detecting abnormal sensors via machine learning: An IoT farming WSN-based architecture case study. Measurement.

[79] Kamizono K., Ikeda K., Kitajima H., Yasuda S., Tanaka T. (2021). FDC Based on Neural Network with Harmonic Sensor to Prevent Error of Robot IEEE Transactions on Semiconductor Manufacturing. IEEE Trans. Semicond. Manuf..

[80] Bidyuk P., Kalinina I., Gozhyj A., Babichev S., Lytvynenko V. (2021). An Approach to Identifying and Filling Data Gaps in Machine Learning Procedures. Lecture Notes in Computational Intelligence and Decision Making. ISDMCI 2021. Lecture Notes on Data Engineering and Communications Technologies.

[81] Kondratenko Y., Kondratenko N. (2018). Real-Time Fuzzy Data Processing Based on a Computational Library of Analytic Models. Data.

[82] Kondratenko Y., Gordienko E. Implementation of the neural networks for adaptive control system on FPGA. Proceedings of the 23th Int. DAAAM Symp. Intelligent Manufacturing and Automation.

[83] Choi W., Heo J., Ahn C. (2021). Development of Road Surface Detection Algorithm Using CycleGAN-Augmented Dataset. Sensors.

[84] Kondratenko Y.P., Kuntsevich V.M., Chikrii A.A., Gubarev V.F. (2021). Advanced Control Systems: Theory and Applications.

[85] Kuntsevich V.M., Gubarev V.F., Kondratenko Y.P., Lebedev D., Lysenko V. (2018). Control Systems: Theory and Applications.

[86] Gerasin O., Kondratenko Y., Topalov A. (2018). Dependable robot’s slip displacement sensors based on capacitive registration elements. Proceedings of the IEEE 9th International Conference on Dependable Systems, Services and Technologies (DESSERT).

[87] Kondratenko Y., Gerasin O., Topalov A. (2016). A simulation model for robot’s slip displacement sensors. Int. J. Comput..

[88] Kondratenko Y.P., Gerasin O.S., Topalov A.M. (2015). Modern Sensing Systems of Intelligent Robots Based on Multi-Component Slip Displacement Sensors. Proceedings of the 2015 IEEE 8th International Conference on Intelligent Data Acquisition and Advanced Computing Systems: Technology and Applications (IDAACS).

[89] Kondratenko Y.P., Kondratenko V.Y., Kondratenko Y., Duro R. (2015). Advanced Trends in Design of Slip Displacement Sensors for Intelligent Robots. Advances in Intelligent Robotics and Collaboration Automation. Series on Automation, Control and Robotics.

[90] Zaporozhets Y.M., Kondratenko Y.P., Shyshkin O.S. (2012). Mathematical model of slip displacement sensor with registration of transversal constituents of magnetic field of sensing element. Tech. Electrodyn..

[91] Kondratenko Y., Shvets E., Shyshkin O. Modern Sensor Systems of Intelligent Robots Based on the Slip Displacement Signal Detection. Proceedings of the 18th Int. DAAAM Symp. Intelligent Manufacturing and Automation.

[92] Kondratenko Y.P. Measurement Methods for Slip Displacement Signal Registration. Proceedings of the Second International Symposium on Measurement Technology and Intelligent Instruments.

[93] Massalim Y., Kappassov Z., Varol H.A., Hayward V. (2021). Robust Detection of Absence of Slip in Robot Hands and Feet. Sensors.

[94] Kondratenko Y., Gerasin O., Kozlov O., Topalov A., Kilimanov B. (2021). Inspection mobile robot’s control system with remote IoT-based data transmission. J. Mob. Multimed..

[95] Kondratenko Y., Zaporozhets Y., Rudolph J., Gerasin O., Topalov A., Kozlov O. (2018). Modeling of clamping magnets interaction with ferromagnetic surface for wheel mobile robots. Int. J. Comput..

[96] Kondratenko Y.Y., Zaporozhets Y., Rudolph J., Gerasin O., Topalov A., Kozlov O. (2017). Features of clamping electromagnets using in wheel mobile robots and modeling of their interaction with ferromagnetic plate. Proceedings of the 9th IEEE International Conference on Intelligent Data Acquisition and Advanced Computing Systems: Technology and Applications (IDAACS).

[97] Taranov M., Rudolph J., Wolf C., Kondratenko Y., Gerasin O. (2017). Advanced approaches to reduce number of actors in a magnetically-operated wheel-mover of a mobile robot. Proceedings of the 2017 13th International Conference Perspective Technologies and Methods in MEMS Design (MEMSTECH).

[98] Kondratenko Y.P., Kozlov O.V., Gerasin O.S., Zaporozhets Y.M. (2016). Synthesis and research of neuro-fuzzy observer of clamping force for mobile robot automatic control system. Proceedings of the 2016 IEEE First International Conference on Data Stream Mining & Processing (DSMP).

[99] Kondratenko Y., Sichevskyi S., Kondratenko G., Sidenko I. (2021). Manipulator’s Control System with Application of the Machine Learning. Proceedings of the 11th IEEE International Conference on Intelligent Data Acquisition and Advanced Computing Systems: Technology and Applications (IDAACS).

[100] Ueda M., Iwata K., Shingu H. Tactile sensors for an industrial robot to detect a slip. Proceedings of the 2nd Int. Symp. on Industrial Robots.

[101] Ueda M., Iwata K. Adaptive grasping operation of an industrial robot. Proceedings of the 3rd Int. Symp. Ind. Robots.

[102] Tiwana M.I., Shashank A., Redmond S.J., Lovell N.H. (2011). Characterization of a capacitive tactile shear sensor for application in robotic and upper limb prostheses. Sens. Actuators A.

[103] Kondratenko Y.P., Kondratenko V.Y., Shvets E.A., Yurish S.Y. (2016). Intelligent Slip Displacement Sensors in Robotics. Sensors, Transducers, Signal Conditioning and Wireless Sensors Networks.

[104] Sheng Q., Xu G.Y., Liu G. (2014). Design of PZT Micro-displacement acquisition system. Sens. Transducers.

[105] Zadeh L.A. (1965). Fuzzy sets. Inf. Control..

[106] Mamdani E.H. (1974). Application of fuzzy algorithm for control of a simple dynamic plant. Proc. Inst. Electr. Eng..

[107] Kondratenko Y.P., Simon D., Zadeh L., Yager R., Shahbazova S., Reformat M., Kreinovich V. (2018). Structural and parametric optimization of fuzzy control and decision making systems. Recent Developments and the New Direction in Soft-Computing Foundations and Applications. Studies in Fuzziness and Soft Computing.

[108] Kondratenko Y.P., Klymenko L.P., Al Zu’bi E.Y.M. (2013). Structural Optimization of Fuzzy Systems’ Rules Base and Aggregation Models. Kybernetes.

[109] Kondratenko Y.P., Altameem T.A., Al Zubi E.Y.M. (2010). The optimisation of digital controllers for fuzzy systems design. Adv. Model. Anal..

[110] Kondratenko Y.P., Al Zubi E.Y.M. The Optimisation Approach for Increasing Efficiency of Digital Fuzzy Controllers. Proceedings of the Annals of DAAAM for 2009 & Proceeding of the 20th Int. DAAAM Symp. Intelligent Manufacturing and Automation.

[111] Kondratenko Y.P., Kozlov A.V. (2019). Parametric optimization of fuzzy control systems based on hybrid particle swarm algorithms with elite strategy. J. Autom. Inf. Sci..

[112] Pedrycz W., Li K., Reformat M., Tamir D., Rishe N., Kandel A. (2015). Evolutionary reduction of fuzzy rule-based models. Fifty Years of Fuzzy Logic and Its Applications. Studies in Fuzziness and Soft Computing.

[113] Christensen L., Fischer N., Kroffke S., Lemburg J., Ahlers R. (2011). Cost-Effective Autonomous Robots for Ballast Water Tank Inspection. J. Ship Prod. Des..

[114] Souto D., Faiña A., Lypez-Peca F., Duro R.J. (2013). Lappa: A new type of robot for underwater non-magnetic and complex hull cleaning. Proceedings of the IEEE International Conference on Robotics and Automation (ICRA).

[115] Ross B., Bares J., Fromme C. (2003). A Semi-Autonomous Robot for Stripping Paint from Large Vessels. Int. J. Robot. Res..

[116] Kondratenko Y., Kozlov O., Gerasin O. (2019). Neuroevolutionary approach to control of complex multicoordinate interrelated plants. Int. J. Comput..

[117] Gerasin O., Kozlov O., Kondratenko G., Rudolph J., Kondratenko Y. (2019). Neural controller for mobile multipurpose caterpillar robot. Proceedings of the 2019 10th IEEE International Conference on Intelligent Data Acquisition and Advanced Computing Systems: Technology and Applications (IDAACS).

[118] Michael S. (2021). Metrological Characterization and Comparison of D415, D455, L515 RealSense Devices in the Close Range. Sensors.

[119] Palar P.S., Vargas Terres V., Oliveira A.S. (2020). Human–Robot Interface for Embedding Sliding Adjustable Autonomy Methods. Sensors.

[120] Atamanyuk I.P., Kondratenko V.Y., Kozlov O.V., Kondratenko Y.P., Engemann K.J., Gil-Lafuente A.M., Merigo J.L. (2012). The Algorithm of Optimal Polynomial Extrapolation of Random Processes. Lecture Notes in Business Information Processing, Proceedings of the International Conference Modeling and Simulation in Engineering, Economics and Management, New Rochelle, NY, USA, 30 May–1 June 2012.

[121] Ryguła A. (2021). Influence of Trajectory and Dynamics of Vehicle Motion on Signal Patterns in the WIM System. Sensors.

[122] Khan F., Ahmad S., Gürüler H., Cetin G., Whangbo T., Kim C.-G. (2021). An Efficient and Reliable Algorithm for Wireless Sensor Network. Sensors.

[123] Yu H., Chen C., Lu N., Lu N., Wang C. (2021). Deep Auto-Encoder and Deep Forest-Assisted Failure Prognosis for Dynamic Predictive Maintenance Scheduling. Sensors.

[124] Atamanyuk I., Kondratenko Y., Shebaninm V., Mirgorod V. (2015). Method of Polynomial Predictive Control of Fail-Safe Operation of Technical Systems. Proceedings of the XIIIth International Conference the Experience of Designing and Application of CAD Systems in Microelectronics (CADSM).

[125] Atamanyuk I., Shebanin V., Kondratenko Y., Volosyuk Y., Sheptylevskyi O., Atamaniuk V. (2019). Predictive Control of Electrical Equipment Reliability on the Basis of the Non-linear Canonical Model of a Vector Random Sequence. Proceedings of the IEEE International Conference on Modern Electrical and Energy Systems (MEES).

[126] Everitt B.S. (2006). The Cambridge Dictionary of Statistics.

[127] Nagrath I.J., Shripal P.P., Chand A. (1995). Development and Implementation of Intelligent Control Strategy for Robotic Manipulator. Proceedings of the IEEE/IAS International Conference on Industrial Automation and Control.

[128] Alagöz Y., Karabayır O., Mustaçoğlu A.F. (2020). Target Classification Using YOLOv2 in Land-Based Marine Surveillance Radar. Proceedings of the 28th Signal Processing and Communications Applications Conference (SIU).

[129] Zhang H., Zhang L., Li P., Gu D. (2018). Yarn-dyed Fabric Defect Detection with YOLOV2 Based on Deep Convolution Neural Networks. Proceedings of the 7th Data Driven Control and Learning Systems Conference (DDCLS).

[130] Wang M., Liu M., Zhang F., Lei G., Guo J., Wang L. (2018). Fast Classification and Detection of Fish Images with YOLOv2. Proceedings of the OCEANS—MTS/IEEE Kobe Techno-Oceans Conference (OTO).

[131] Li L., Chou W., Zhou W., Lou M. (2016). Design Patterns and Extensibility of REST API for Networking Applications. IEEE Trans. Netw. Serv. Manag..

[132] Rivera S., Iannillo A.K., Lagraa S., Joly C., State R. (2020). ROS-FM: Fast Monitoring for the Robotic Operating System (ROS). Proceedings of the 25th International Conference on Engineering of Complex Computer Systems (ICECCS).

[133] Görner M., Haschke R., Ritter H., Zhang J. (2019). MoveIt! Task Constructor for Task-Level Motion Planning. Proceedings of the International Conference on Robotics and Automation (ICRA).

[134] Deng H., Xiong J., Xia Z. (2017). Mobile Manipulation Task Simulation using ROS with MoveIt. Proceedings of the IEEE International Conference on Real-time Computing and Robotics (RCAR).

[135] Youakim D., Ridao P., Palomeras N., Spadafora F., Ribas D., Muzzupappa M. (2017). MoveIt!: Autonomous Underwater Free-Floating Manipulation. IEEE Robot. Autom. Mag..

[136] Salameen L., Estatieh A., Darbisi S., Tutunji T.A., Rawashdeh N.A. (2020). Interfacing Computing Platforms for Dynamic Control and Identification of an Industrial KUKA Robot Arm. Proceedings of the 21st International Conference on Research and Education in Mechatronics (REM).

[137] Gugnani S., Lu X., Panda D.K. (2017). Swift-X: Accelerating OpenStack Swift with RDMA for Building an Efficient HPC Cloud. Proceedings of the 17th IEEE/ACM International Symposium on Cluster, Cloud and Grid Computing (CCGRID).

